# Acute Coronary Syndrome Risk Prediction Using Portable Cable-Free ECG Device Combined With Clinical Risk Assessment

**DOI:** 10.1016/j.jacadv.2026.102827

**Published:** 2026-05-21

**Authors:** Alexei Shvilkin, Sanja Zdolšek, Nataša Zlatić, Dario Jelić, Vladimir A. Atanasoski, Marjan N. Miletić, Boško P. Bojović, Ljupčo R. Hadžievski, Peter J. Zimetbaum, C. Michael Gibson, Vladan Vukčević

**Affiliations:** a Cardiovascular Division, Department of Medicine, Beth Israel Deaconess Medical Center, Boston, Massachusetts, USA; b Vinča Institute of Nuclear Sciences, University of Belgrade, Belgrade, Serbia; c Department of Medicine, Cardiology Clinic, Clinical Center of Serbia, Belgrade, Serbia; d Baim Institute for Clinical Research, Boston, Massachusetts, USA

**Keywords:** acute coronary syndrome, mobile health, portable electrocardiography, pre-hospital triage, risk stratification

## Abstract

**Background:**

Patient uncertainty in symptom interpretation during acute coronary syndrome (ACS) contributes to patient-related presentation delays, increasing morbidity and mortality. Integrating clinical context with electrocardiogram (ECG) analysis in a portable patient-operated ACS risk assessment device may facilitate early self-triage.

**Objectives:**

The objective of the study was to evaluate the diagnostic performance of an algorithm combining pre-existing atherosclerotic cardiovascular risk (PER), symptom risk (SR), and portable ECG recorded using a hand-held cable-free device for ACS risk estimation.

**Methods:**

This prospective single-center study enrolled 212 consecutive emergency department patients with chest pain; 184 patients with complete data were analyzed (96 learning set and 88 test set). Three independent components: PER, SR, and ST-vector loop parameters, derived from a three-lead cable-free credit card-size ECG device were integrated into a hierarchical fusion ACS risk assessment model. In 98 patients, asymptomatic postevent reference portable ECG was obtained 9 to 12 months later. Algorithm performance was evaluated using receiver operating characteristic curve analysis and compared with human consensus interpretation of 12-lead ECG plus PER and SR information.

**Results:**

Algorithm ACS prediction using a single portable ECG achieved area under the curve of 0.865, increasing to 0.929 with postevent reference comparison (*P* = 0.036). At matched sensitivity, the model specificity was comparable to the human consensus (false-positive rate 0.527 vs 0.458; *P* = 0.709) and improved using reference comparison (model false-positive rate 0.198 vs human 0.556; *P* = 0.004).

**Conclusions:**

An algorithm combining clinical risk factors, symptoms, and ECG recording using hand-held cable-free device provides clinically meaningful ACS risk stratification. If fully integrated into a personal device, it may help support early self-triage decisions and reduce patient-related prehospital delays.

Patient-related symptom-to-treatment delays in seeking care for chest pain^1^ represent a major component of prehospital delays and contribute substantially to out-of-hospital cardiac arrest and prolonged symptom-to-treatment times in acute coronary syndrome (ACS),^2^, ^3^, ^4^ with little improvement over recent decades.^5^

Several prehospital electrocardiogram (ECG)-based strategies have been used to shorten symptom-to-treatment time, including transmission of a prehospital 12-lead ECG,^6^ implantable intracardiac ST-segment monitoring,^7^ and personal portable ECG recording systems.^8^^,^^9^

Transmission of a 12-lead ECG requires specialized equipment and trained personnel, and other approaches rely on lead-based systems or intracardiac implants, which may limit their practicality.

Furthermore, the increase in the prevalence of non-ST-elevation myocardial infarction (NSTEMI)^10^ and the dependence of diagnostic ECG capabilities on the conditional probability of the disease^11^ limits the value of ECG-only ACS detection approaches.

We hypothesized that an algorithm combining portable ECG recording using a cable-free self-operated hand-held device with independent clinical ACS risk predictors would enable accurate ACS risk estimation. If integrated within a portable personal smartphone-connected device, such approach could support early patient decision-making, reduce patient-related prehospital delays, and limit unnecessary emergency department utilization among low-risk (“worried well”) patients.

## Objective

To assess the feasibility of an ACS risk prediction algorithm combining pre-existing atherosclerotic cardiovascular disease (ASCVD) risk (PER), symptom risk (SR), and portable three-lead ECG recording to estimate ACS risk in patients with chest pain.

## Methods

### Patients

This prospective, single-center study enrolled consecutive adult patients presenting with nontraumatic chest pain warranting evaluation for ACS to the Emergency Department of the Clinical Centre of Serbia, Belgrade, a tertiary ACS referral center. Patients were either transported by ambulance with suspected ACS or referred to the chest pain unit by the emergency department triage nurses when initial evaluation did not identify an obvious noncardiac cause (eg, chest trauma, pneumonia, or other alternative diagnoses). Inclusion and exclusion criteria are provided in the Supplemental Table 1. The study was approved by the institutional ethics committee, and all participants provided written informed consent.

All patients were managed with observation, serial ECG recordings, and high sensitivity troponin testing as indicated according to the local chest pain protocol. Patients presenting with ST-segment elevation myocardial infarction (STEMI) were treated with percutaneous coronary intervention. Stress testing and nonemergent cardiac catheterization were performed before discharge at the discretion of the attending physician.

### ACS risk model components

The ACS risk model estimates ACS probability by combining 3 independent predictor families.

**Figure 1:**
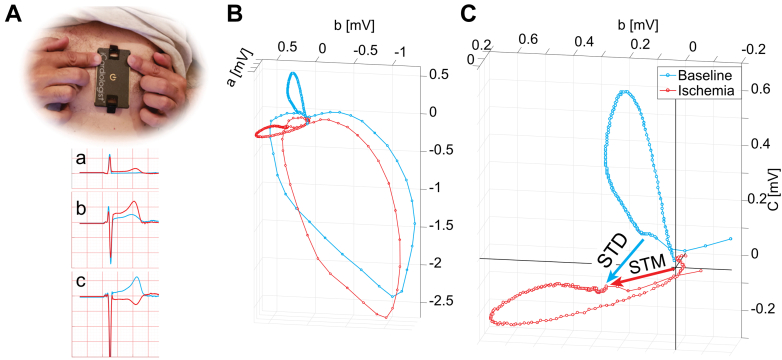
The HeartBeam Device (A) A cell phone connected cable-free device records a 30-second 3-lead ECG with leads a, b, and c, approximately corresponding to leads I, aVF, and V_2_, using 2 finger and 2 integrated chest electrodes. (B) It is transformed into a three-dimensional QRST-vector loops. (C) ST-segment measurements (presented on magnified baseline and ischemic recordings T-wave loops). STM = ST-vector magnitude; STD = ST-vector difference from reference recording, as previously described.^16^ Dots represent 2-ms timing markers.

Adapted from Shvilkin et al.^16^

1) PER, derived from demographic and medical history variables;
2) SR, calculated using prognostically significant symptoms obtained by a proprietary symptom questionnaire based on prior literature (Supplemental Table 2);^12^, ^13^, ^14^
3) Three-lead 30-second hand-held ECG recording obtained using the HeartBeam device (HB-ECG). The HeartBeam device is a portable credit card-sized cable-free three-lead ECG recorder with U.S. Food and Drug Administration clearance for 12-lead ECG synthesis for arrhythmia detection (510 [k] clearance K231424).^15^ The device incorporates 4 electrodes (2 finger electrodes and 2 spring-loaded chest electrodes). HB-ECG is recorded by applying the device to the chest, generating 3 noncoplanar leads (a, b, and c) forming a 3-dimensional vector system with axes approximately corresponding to leads I, aVF, and V_2_ (Figure 1).

The HeartBeam device was used solely for acquisition of the HB-ECG signal, whereas PER and SR variables were collected electronically by study physicians using standardized questionnaires and medical records information.

### Study procedures

Designated research physicians trained in study procedures and questionnaire administration, and not directly involved in patient care, were present in the chest pain unit 24 hours per day to ensure enrollment of consecutive eligible patients.

After receiving informed consent, study physicians administered a structured symptom questionnaire for SR estimation and collected demographic and clinical history variables required for calculation of PER in parallel with the initial patient evaluation and subsequent medical record review. The symptom questionnaire was originally developed in English, translated into the Serbian, and administered using standardized wording of the original questions. Study physicians supervised and assisted patients with acquisition of the HB-ECG, obtained nearly simultaneously with standard 12-lead ECG recordings. Data were entered electronically into case report forms, which were subsequently updated with follow-up clinical information and the final discharge diagnosis.

In a subset of geographically available patients (willing to come to the clinic or accessible for a home visit), an asymptomatic HB-ECG recording 9 to 12 months after the index admission was obtained as “postevent reference.”

### Outcome definition

Clinical outcomes were adjudicated by the Clinical Events Committee based on all available data and classified as ACS, defined according to the Fourth Universal Definition of Myocardial Infarction,^17^ including STEMI, NSTEMI, and unstable angina, or as noncardiac chest pain.

### Study endpoints

The primary endpoint of the study was the diagnostic accuracy of the ACS risk prediction model in ACS detection.

The secondary endpoints of the study were:

1. Diagnostic performance of ACS risk prediction model for STEMI detection.
2. Human comparison (ECG interpretation): Diagnostic performance of HB-ECG-only–based ACS assessment vs five-cardiologist consensus interpretation of the index 12-lead ECG.
3. Human comparison (clinical triage assessment): Performance of ACS risk prediction model vs the consensus decision of 3 chest pain unit physicians using the same clinical inputs (PER and SR information) plus the index 12-lead ECG.

### Rationale and structure of the fusion ACS risk prediction model

The algorithm modeling strategy was inspired by the diagnostic logic of the Diamond-Forrester serial likelihood ratio analysis,^11^ in which independent predictors are combined sequentially to refine disease probability. Each family of predictors—PER, SR, and HB-ECG parameters—was evaluated and modeled separately as independent components for the final fusion model. Multiple component models were tested, and the best performing models based on receiver operating characteristic (ROC) curve analysis were combined in the fusion model as described subsequently.

### SR model

Chest pain features were characterized across 9 clinically relevant dimensions including location, characteristics, area size, radiation, duration, intensity, associated symptoms, and reproducibility (see Supplemental Table 2). The best-performing SR-only ACS prediction model was a weighted logistic regression using 7 prespecified positive and negative predictors selected based on clinical relevance and univariate ORs: pain intensity (on ordinal scale 0-10), inverse U-shaped duration score (very short and very long symptom duration assigned lower weights), larger pain area size, arm radiation, burning/aching quality (positively associated with ACS), and pleuritic character, stabbing/pinprick quality (negatively associated with ACS). The SR model generated a predicted probability of ACS between 0 and 1. Univariate ORs of symptom features presented in Supplemental Table 3.

### PER model

PER model incorporated baseline demographic and clinical history variables that were obtainable in all patients at presentation or by chart review. Variables showing univariate association with ACS included age, sex, diabetes, chronic aspirin use, and smoking status (Supplemental Table 4). The model was implemented as a tree-based gradient boosting machine with logistic loss, tuned by internal cross-validation providing outputs of predicted ACS probability ranging from 0 to 1.

### HeartBeam ECG analysis

HeartBeam ECG measurements are based on a 3-dimensional analysis of the initial portion of the ST-vector loop, producing 2 parameters: absolute ST-vector magnitude (STM) and ST-vector difference from reference recording (STD) from the reference recording as previously described^16^ (Figure 1C).

For STD measurement we used postevent reference HB-ECG as an individualized comparator intended to reduce the influence of pre-existing patient-specific ECG abnormalities. Because such recordings may be influenced by interval clinical events, additional validation analyses were performed in this subset, including assessment of outcome differences according to reference availability, sensitivity analyses stratified by interim clinical event categories, and permutation analysis.

### Final fusion ACS prediction models

Final fusion models were constructed by combining the independently derived PER and SR component model probabilities with HB-ECG parameters in a ridge-regularized logistic regression model. Specifically, the predicted probabilities generated by the PER and SR models were entered as continuous predictors together with STM for full-cohort analyses and with STM and/or STD for postevent reference analyses as direct values in mV. A clinical-only fusion model combining PER and SR probabilities was also evaluated. Model coefficients were estimated in the learning set and then applied without modification to the test set. Separate fusion models were developed for ACS and STEMI prediction.

### Human comparison

Human assessment was designed to approximate physician decision-making in an early triage or prehospital setting using a single admission 12-lead ECG. Two blinded assessment modes were evaluated. In the ECG-only mode, a panel of 5 cardiologists (interventional cardiologists/electrophysiologists) reviewed the ECG without clinical context and provided binary ACS (yes/no) interpretations. In the clinical triage mode, a separate panel of 3 chest pain unit physicians, not involved in study patient care, evaluated the same ECG together with SR and PER inputs including symptom features and baseline cardiovascular risk factors used in the algorithm, to determine the presence of ACS (yes/no). Consensus classification (majority vote) was used for all human-ACS risk model analyses.

### Statistical analysis

Descriptive comparisons between the learning and test cohorts were performed using standard parametric and nonparametric methods. Continuous variables were presented as mean ± SD when normally distributed and as median (IQR) otherwise. Continuous variables were compared using 2-sample t-tests or Wilcoxon rank sum tests, respectively. Categorical variables were presented as counts (percentages) and were compared using chi-square or Fisher exact tests as appropriate. A 2-sided *P* value <0.05 was considered statistically significant.

### Model development, validation, and comparisons

Model performance was assessed using ROC analysis, with area under the curve (AUC) estimates and 95% CIs obtained via nonparametric bootstrapping. When comparing AUCs from models evaluated on the same cohort and outcome, pairwise statistical comparisons were performed using the DeLong method. Multicollinearity among candidate predictors was assessed using pairwise Spearman correlation analysis and variance inflation factors (VIFs), with VIF values <2.0 considered indicative of low collinearity.

### Comparison of ACS risk prediction models with human readers

Individual and consensus human performance were presented as single operating points of sensitivity/specificity in the ROC space. Model-to-human comparisons were made based on false-positive rate (FPR) at matched to the human consensus sensitivity. Differences were summarized as: ΔFPR = FPR_human − FPR_model, where model FPR was estimated as the bootstrap median across resampled test sets, with 95% CIs and *P* values obtained by paired bootstrap resampling.

All data analysis was performed using MATLAB (version 2023a; MathWorks).

## Results

### Study enrollment and clinical outcomes

A total of 212 patients were enrolled and assigned consecutively to learning (n = 112) and test (n = 100) cohorts. Twenty-eight patients were excluded (missing clinical data or 12-lead ECGs - 18, inadequate quality HB-ECG - 10); 184 patients with complete data were included in the analysis (learning set n = 96; test set n = 88). Postevent reference ECG recordings were available in 51 and 47 patients, respectively (*P* = 1.000).

**Figure 2:**
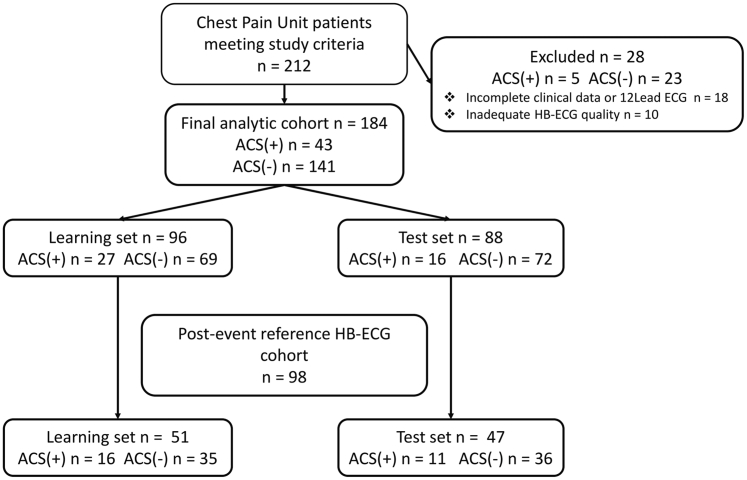
Study Flowchart ACS = acute coronary syndrome; HB-ECG = HeartBeam electrocardiogram.

ACS was diagnosed in 43 of 184 patients (23%), including 29 NSTEMI and 14 STEMI cases. ACS prevalence was 28.1% in the learning cohort and 18.2% in the test cohort (*P* = 0.111). Study flowchart is presented in Figure 2. Baseline clinical characteristics were similar between the learning and test cohorts. Detailed baseline characteristics and clinical outcomes are summarized in Table 1.

**Table 1:** Baseline Clinical Characteristics and Outcomes

	Learning Set (n = 96)	Test Set (n = 88)	*P* Value
Sex (male)	48 (50.0%)	40 (45.5%)	0.560
Age, years	58.01 ± 14.51	56.07 ± 14.93	0.370
Weight, kg	80.00 (67.8-90.0)	78.00 (70.0-89.3)	1.000
Body mass index, kg/m^2^	26.54 (24.3-29.1)	26.96 (24.4-29.4)	0.815
Current smoker	33 (34.4%)	43 (48.9%)	0.050
Former smoker	20 (20.8%)	21 (23.9%)	0.720
Any smoker (current or former)	52 (54.2%)	63 (71.6%)	0.020
Hyperlipidemia	43 (44.8%)	33 (37.5%)	0.370
Diabetes mellitus	19 (19.8%)	11 (12.5%)	0.230
Hypertension	51 (53.1%)	51 (58.0%)	0.550
History of CHD	43 (44.8%)	28 (31.8%)	0.071
Angina	30 (31.2%)	25 (28.4%)	0.674
Myocardial infarction	21 (21.9%)	16 (18.2%)	0.532
Stents	16 (16.7%)	12 (13.6%)	0.568
Coronary artery bypass graft	4 (4.2%)	0 (0.0%)	0.122
Chronic NTG use	31 (32.3%)	22 (25.0%)	0.330
Peripheral vascular disease	13 (13.5%)	13 (14.8%)	0.811
Stroke/TIA	7 (7.3%)	7 (8.0%)	1.000
ASCVD	52 (54.2%)	36 (40.9%)	0.072
Pain intensity (0-10)	6 (5-8)	6 (5-8)	0.760
STM, mV	0.12 (0.07-0.18)	0.10 (0.07-0.18)	0.280
STD, mV	0.07 (0.05-0.11)	0.08 (0.04-0.13)	0.840
ACS	27 (28.1%)	16 (18.2%)	0.111
NSTEMI	18 (18.8%)	11 (12.5%)	0.310
STEMI	9 (9.4%)	5 (5.7%)	0.420
Cath lab activation	30 (31.2%)	15 (20.3%)	0.108
Emergent percutaneous coronary intervention	22 (22.9%)	10 (13.5%)	0.120

Continuous variables are presented as mean ± SD or median (IQR), as appropriate; categorical variables are presented as n (%).

ACS = acute coronary syndrome; ASCVD = atherosclerotic cardiovascular disease; CHD = coronary heart disease; NTG = nitroglycerin; NSTEMI = non-ST-segment elevation myocardial infarction; STD = ST-vector difference from reference recording; STEMI = ST-segment elevation myocardial infarction; STM = ST-vector magnitude; TIA = transient ischemic attack.

### Component and full fusion model ACS and STEMI prediction performance

Because some subgroup analyses were based on relatively small numbers of ACS and especially STEMI events, model performance estimates had wide, often overlapping CIs (Table 2). Accordingly, between-model differences are presented as numerical trends unless confirmed by formal pairwise comparison.

**Table 2:** Model Performance for ACS and STEMI Detection

	AUC Learning Set	AUC Test Set	95% CI (Test Set)
ACS: Full cohort - learning (n = 96), test (n = 88)			
Single-predictor models			
PER	0.903	0.796	(0.679, 0.899)
SR	0.816	0.738	(0.591, 0.861)
HB-ECG: STM	0.658	0.789	(0.673, 0.892)
Fusion models			
PER + SR	0.950	0.854	(0.744, 0.943)
PER + SR + STM	0.948	0.865	(0.758, 0.949)
ACS: Postevent reference cohort- learning (n = 51), test (n = 47)			
Single-predictor models			
PER	0.928	0.710	(0.549, 0.858)
SR	0.846	0.736	(0.537, 0.912)
HB-ECG: STM	0.682	0.798	(0.652, 0.917)
HB-ECG: STD	0.764	0.861	(0.662, 1.000)
Fusion models			
PER + SR	0.970	0.788	(0.621, 0.919)
PER + SR + STM	0.968	0.859	(0.725, 0.957)
PER + SR + STD	0.986	0.929	(0.843, 0.988)
STEMI: Full cohort - learning (n = 96), test (n = 88)			
Single-predictor models			
PER	0.964	0.748	(0.504, 0.936)
SR	0.854	0.723	(0.358, 0.982)
HB-ECG: STM	0.736	0.973	(0.921, 1.000)
Fusion models			
PER + SR	0.994	0.745	(0.353, 0.997)
PER + SR + STM	0.994	0.783	(0.482, 1.000)
STEMI: Postevent reference cohort - learning (n = 51), test (n = 47)			
Single-predictor models			
PER	0.948	0.602	(0.293, 0.878)
SR	0.824	0.576	(0.178, 0.978)
HB-ECG: STM	0.670	1.000	(1.000, 1.000)
HB-ECG: STD	0.852	0.985	(0.935, 1.000)
Fusion models			
PER + SR + STM	1.000	0.333	(0.000, 1.000)
PER + SR + STD	0.996	0.636	(0.239, 1.000)

Event counts - ACS: Full cohort 43 cases (learn 27; test 16); postevent reference cohort (learn 16, test 11). STEMI: Full cohort 14 cases; postevent reference cohort 8 cases (learning 5, test 3).

AUC differences between models were not formally compared across all combinations because CIs were wide and largely overlapping; selected pairwise comparisons are reported in the Results section where relevant.

ACS = acute coronary syndrome; AUC = area under the curve; ECG = electrocardiogram; PER = pre-existing ASCVD risk; SR = symptom risk; other abbreviations as in Table 1.

### ACS prediction

**Figure 3:**
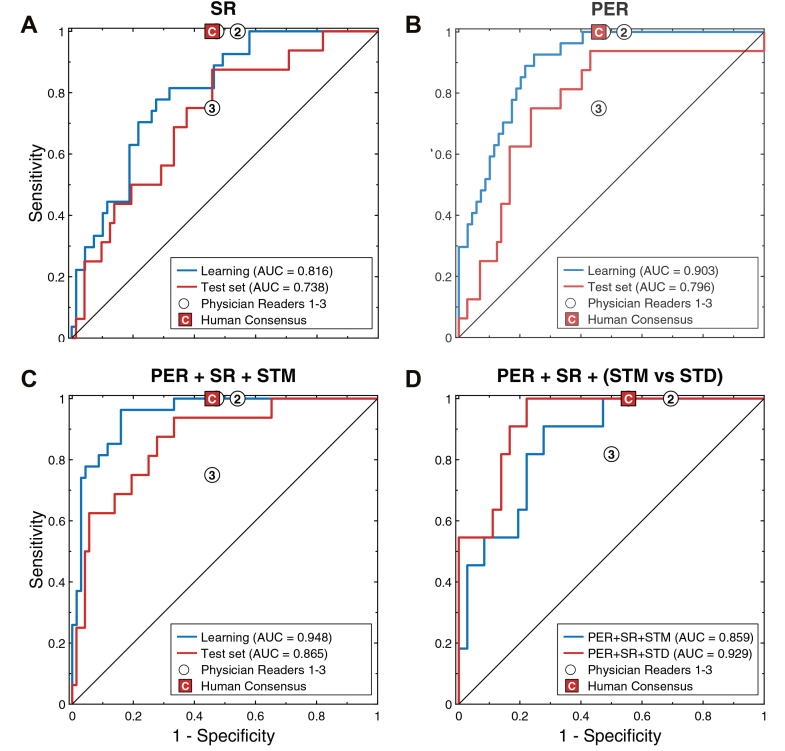
Receiver Operating Characteristic Curves for Acute Coronary Syndrome Prediction Using Component and Fusion Models Receiver operating characteristic (ROC) curves for ACS prediction using symptom risk (SR), pre-existing risk factor (PER), and fusion models incorporating HB-ECG-derived ST metrics. (A to C) Demonstrate model behavior in full-cohort learning and test sets for the SR-only, PER-only, and fusion models, respectively. (D) Shows direct comparison of the PER + SR + STM and PER + SR + STD models in the postevent reference cohort test set. Individual and consensus human clinical triage operating points are shown for reference. In the full test cohort, model false-positive rates at matched human sensitivity were not significantly different from human consensus, whereas in the postevent reference test cohort the PER + SR + STD model showed a significantly lower false-positive rate than human consensus (*P* = 0.004). AUC = area under the receiver-operating characteristic curve; STD = ST-vector difference from reference recording; STM = ST-vector magnitude.

**Figure 4:**
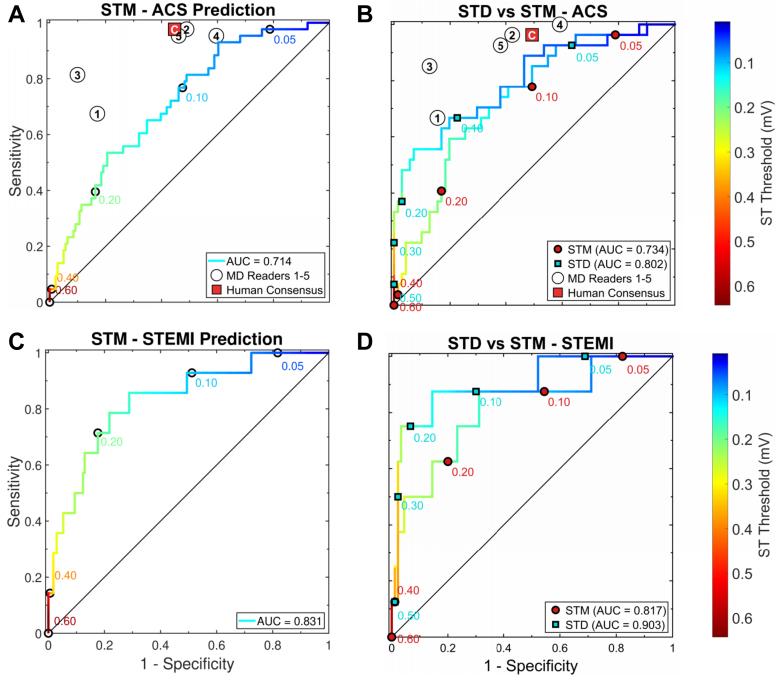
HeartBeam Electrocardiogram-Only Models of Acute Coronary Syndrome and ST-Elevation Myocardial Infarction Prediction Receiver-operating-characteristic (ROC) curves for HB-ECG-only prediction of (A and B) ACS and (C and D) STEMI. Panels A and C show STM-based prediction in the full cohort (n = 184). Panels B and D show direct comparison of STM and STD in the post-event reference cohort (n = 98). Line colors and threshold-labeled points indicate ST-segment thresholds (mV). Individual human ECG-only reader operating points (readers 1-5) and human consensus (C) are shown for ACS only. In the postevent reference cohort, STD yielded numerically higher AUCs than STM for both ACS and STEMI, although these differences were not statistically significant. AUC values for each model are shown in the panel legends. Abbreviations as in Figures 2 and 3.

Component models based on individual predictor families—PER, SR, and HB-ECG—metrics each provided independent discriminatory signals for ACS, although none alone matched the performance of the fusion models. In the full test cohort, the PER, SR, and STM component models achieved AUC values of 0.796, 0.738, and 0.789, respectively (Table 2, Figures 3A and 3B, Figure 4A). Numerically, discrimination increased with integration of multiple predictor families, with SR + PER reaching an AUC of 0.854 and SR + PER + STM an AUC of 0.865 (Table 2, Figure 3C). In the postevent reference test cohort, the STD-only model achieved an AUC of 0.861 compared with 0.798 for STM, and the SR + PER + STD fusion model showed the highest observed AUC = 0.929 compared with AUC = 0.859 for SR + PER + STM (Table 2). Visual comparison of STM- and STD-based models in the test postevent reference cohort (Figure 3D) suggests improved discrimination at both ends of the ROC curve (rule-in and rule-out advantage) with STD-based referencing.

### STEMI prediction

For STEMI, HB-ECG–based models showed the highest numerical discrimination, reflecting the central role of ST-segment shift in the identification of acute coronary occlusion. In the full test cohort, the STM model achieved an AUC of 0.973, exceeding the corresponding fusion models incorporating PER and SR (Table 2). In the postevent reference setting, both STM and STD demonstrated very high discrimination in the test cohort (AUC: 1.000 for STM and 0.985 for STD). Visual inspection of the ROC curves in Figure 3D suggests that STD-based referencing may provide improved specificity in the presence of large baseline ST-segment deviations, although CIs were wide and event counts low.

### Model consistency and internal validity assessment

Within the SR predictor family, statistical multicollinearity was minimal (all VIFs <2.0). The only notable association was a modest correlation between pleuritic pain and smaller pain area (Spearman ρ ≈ 0.24), reflecting related characteristics of musculoskeletal chest pain (Supplemental Figure 1, Supplemental Table 5).

Within the PER predictor family, correlations were observed among closely related coronary disease history variables (eg, prior myocardial infarction, coronary revascularization, angina, and use of aspirin or nitroglycerin). Of these, only aspirin use remained in the final PER model, and overall multicollinearity of final predictors was low (VIF: 1.02-1.26) (Supplemental Figure 2, Supplemental Table 5).

Between predictor families, correlations were weak (|Spearman ρ| ≤0.29), indicating that symptom characteristics and clinical risk factors contributed largely independent information (Supplemental Figure 3). Collectively, these findings support complementary and largely independent contributions of SR and PER predictors.

For ACS prediction, calibration analyses demonstrated good agreement between predicted and observed event rates across the range of estimated risk for both component models and the final fusion model, supporting internal validity. In contrast, STEMI models demonstrated inconsistent calibration and wide CIs consistent with less predictive values and low event counts (Supplemental Figures 4 to 7).

### Postevent reference HB-ECG validation

Patients with and without available postevent reference HB-ECG did not differ significantly in rates of ACS (27.6% vs 18.6%; *P* = 0.166) or STEMI (8.2% vs 7.0%; *P* = 0.789), arguing against major selection bias related to reference availability (Supplemental Table 6).

Among the 98 patients with postevent reference HB-ECG, 47 had interval clinical events (symptomatic ambulatory visits or admissions), categorized as major cardiac events, (coronary artery bypass grafting, percutaneous coronary intervention, and unstable angina), n = 8, nonmajor cardiac events (angina, hypertension, and arrhythmias), n = 13, and noncardiac events, n = 22 (Supplemental Table 7).

**Figure 5:**
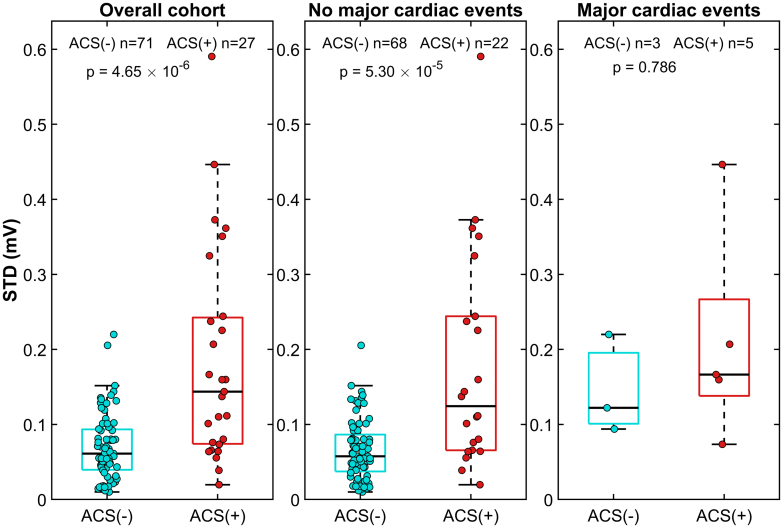
ST-Segment Shift Difference by Acute Coronary Syndrome Status Stratified by Follow-Up Events ST-vector difference from reference recording (STD) by acute coronary syndrome (ACS) status in the postevent reference cohort. Left, overall cohort; middle, patients without major cardiac events during follow-up; right, patients with major cardiac events during follow-up. Box-and-whisker plots with overlaid individual observations are shown for ACS-negative and ACS-positive patients. Boxes indicate the IQR with median lines; whiskers extend to 1.5 × the IQR. STD was higher in ACS-positive patients in the overall cohort and in those without major cardiac events, whereas no significant difference was observed in the small subgroup with major cardiac events. This pattern is consistent with conservative bias in STD estimates in patients with subsequent major cardiac events. Abbreviations as in Figures 2 and 3.

Across all postevent follow-up strata, ACS-positive STD values remained stable (mean 0.153-0.196). In all strata except the major cardiac events group ACS-negative STD values remained substantially lower (mean 0.066-0.083; *P* = 1.09 × 10^−5^ to 0.012), with corresponding consistent ACS discrimination (AUC: 0.772-0.814). In the major cardiac events subgroup, ACS-negative STD was close to ACS-positive range (0.153 ± 0.064 vs 0.169 ± 0.077; *P* = 0.786) (Figure 5), resulting in a conservative reduction in group separation and lower AUC (0.597). Across all strata, including major cardiac events, STD AUC was numerically higher than STM (Supplemental Tables 8 and 9).

To further evaluate the contribution of patient-specific referencing, we performed a permutation analysis in which postevent reference HB-ECGs were randomly reassigned across patients while preserving the original event HB-ECG vectors and STM values. Random reassignment increased STD predominantly in ACS-negative patients, whereas the effect was smaller and not statistically significant in ACS-positive patients (Supplemental Figure 9). Patient-matched STD demonstrated the highest numerical discrimination for ACS (AUC: 0.815) compared with randomly paired STD (AUC: 0.751) and STM (AUC: 0.735) (Supplemental Tables 10 and 11). Although pairwise AUC differences did not reach statistical significance, several consistent findings, including the absence of selection bias, stability of STD across follow-up event strata, conservative distortion limited to the major-cardiac-event subgroup, and a numerical advantage of patient-matched over randomly paired STD support the use of postevent HB-ECG as an individualized physiological reference.

### Human ECG-only interpretation for ACS

ECG-only human interpretation demonstrated high sensitivity with modest specificity (consensus sensitivity 0.953; specificity 0.617), consistent with a conservative emergency department diagnostic strategy prioritizing avoidance of missed ACS (Figures 4A and 4B). Individual reader sensitivity ranged from 0.674 to 0.977 and specificity from 0.404 to 0.901. Overall interobserver agreement was fair-to-moderate (Krippendorff’s α = 0.380), with pairwise reader agreement ranging from κ = 0.237 to 0.809 and agreement between individual readers and the consensus ranging from κ = 0.443 to 0.781 (Supplemental Table 12, Supplemental Figure 10).

### Human clinical triage assessment (12-lead ECG + SR + PER)

When human binary clinical assessment for ACS was performed using the admission 12-lead ECG together with ASCVD risk factors and symptom information, overall interobserver agreement in the 3-physician panel was moderate (Krippendorff’s α = 0.511) and higher than that observed in the separate ECG-only interpretation panel (α = 0.380). Consensus sensitivity reached 100% with specificity of 0.542 in the test cohort (Figures 3 and 4, Supplemental Table 13).

### Comparison of ACS risk prediction models with human clinical triage assessment

At the matched human sensitivity (100%), ACS risk prediction models based on STM alone and on clinical-ECG fusion models demonstrated numerically higher but statistically comparable FPRs relative to the human clinical triage consensus in the full test cohort (human FPR 0.458) (Table 3). Specifically, the STM-only model showed an FPR of 0.531 (*P* = 0.496), the SR + PER model an FPR of 0.575 (*P* = 0.559), and the SR + PER + STM fusion model an FPR of 0.527 (*P* = 0.709). In contrast, in the postevent reference cohort, the SR + PER + STD model achieved a significantly lower FPR than the human consensus (model FPR 0.198 vs human FPR 0.556; *P* = 0.004) (Table 3, Figures 3 and 4).

**Table 3:** Comparison of ACS Risk Prediction Models With Human Consensus at Matched Sensitivity (100%) in the Test Set Full Cohort (N = 88; ACS = 16) and Postevent Reference Subset (N = 47; ACS = 13)^a^

Predictors	Cohort	Human FPR	Model FPR	ΔFPR (Human - Model)	95% CI	*P* Value
HB-ECG: STM	Full cohort	0.458	0.531	−0.073	(−0.261, 0.170)	0.496
SR + PER	Full cohort	0.458	0.575	−0.117	(−0.380, 0.276)	0.559
SR + PER + STM	Full cohort	0.458	0.527	−0.069	(−0.333, 0.283)	0.709
HB-ECG: STD	Post-event reference	0.556	0.658	−0.102	(−0.478, 0.564)	0.720
SR + PER	Post-event reference	0.556	0.591	−0.035	(−0.353, 0.397)	0.870
SR + PER + STD	**Post-event reference**	**0.556**	**0.198**	**0.358**	**(0.108, 0.594)**	**0.004**

**Bold** values indicate statistically significant differences, *P* < 0.05.

FPR = false-positive rate; other abbreviations as in Tables 1 and 2.

a ΔFPR values are bootstrap-based median estimates at matched human sensitivity and may not correspond to a single empirical point on the ROC curves.

## Discussion

### ACS risk prediction model structure: hierarchical fusion model

In this study, we demonstrate proof of concept that a hierarchical ACS prediction model combining independent families of clinical and ECG features achieves clinically meaningful diagnostic performance in chest pain evaluation. It is grounded in Bayesian diagnostic reasoning and follows the Diamond-Forrester concept of serial likelihood ratio analysis, in which ECG interpretation can be vastly different depending on the combination of independent clinical context variables.^11^ The same logic is consciously or intuitively applied during outpatient chest pain physician evaluation,^18^, ^19^, ^20^ where ASCVD risk and symptom define baseline likelihood, and ECG-derived features act as conditional modifiers. Central Illustration summarizes this concept: independent PER and SR values create a combined pretest risk plane greatly improving separation of otherwise overlapping STMs.

**Central Illustration:**
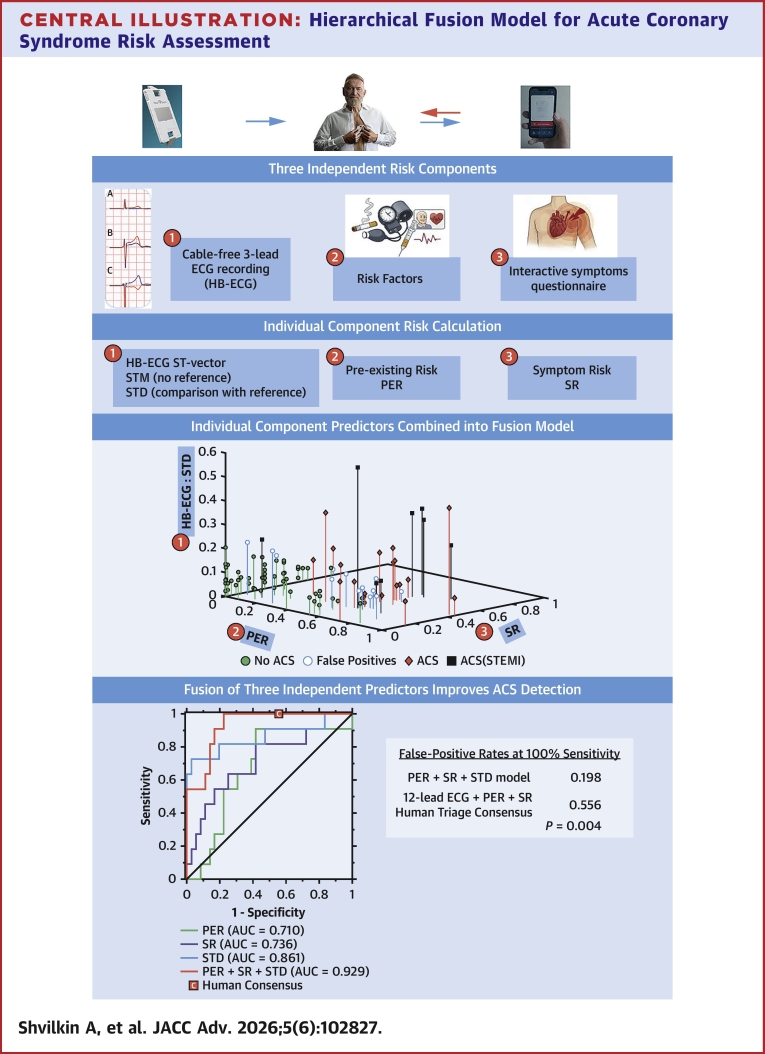
Hierarchical Fusion Model for Acute Coronary Syndrome Risk Assessment Three independent risk components: pre-existing risk factors, symptom questionnaire, and HB-ECG recording are collected to generate component risk estimates: pre-existing risk (PER), symptom risk (SR), and HB-ECG ST-vector metrics (ST-vector magnitude [STM] and ST-vector difference from reference recording [STD] from postevent reference). These predictors are then integrated into a fusion model that stratifies ACS likelihood in a multidimensional risk space. Fusion models incorporating STD and STM are compared with consensus human diagnosis, with the STD-based model demonstrating superior diagnostic performance. ACS = acute coronary syndrome; HB-ECG = HeartBeam electrocardiogram; STEMI = ST-segment elevation myocardial infarction.

In this regard, our algorithm differs from the ECG-only ischemia detection methods that are inherently limited in ACS detection since many events—particularly NSTEMI and unstable angina—can occur with minimal or absent ECG changes.

Despite limited data availability (lack of certain PER components required for use in standard ASCVD risk assessment tools, artificial “postevent reference” recordings), both single component and full models demonstrated consistent internal behavior. Importantly, the final model provided useful discrimination at both extremes of the ROC space, supporting its use in both rule-in (high sensitivity at 100% specificity) and rule-out (high specificity at 100% sensitivity) scenarios (Figure 4D).

Our exploratory analyses evaluated the potential value of individualized ECG referencing using postevent HB-ECG. Although not representing a true preevent baseline, patient-matched referencing consistently showed numerically better discrimination than nonreferenced ST-vector magnitude. This approach is physiologically plausible especially in ACS-negative patients, in whom ST-segment remains relatively stable over time in the absence of cardiac events, making postevent recordings a reasonable approximation of baseline configuration. In the small subgroup with major interval cardiac events, ACS-negative STD values increased toward the ACS-positive range, reducing group separation and lowering AUC, an effect that would be expected to underestimate rather than inflate model performance. Together, these findings suggest that postevent HB-ECG recording may serve as a useful individual reference, warranting further study with careful analysis of follow-up clinical events.

Although a number of diagnostic scores for higher risk patients presenting with chest pain to the emergency department typically include serial biomarker analysis,^21^^,^^22^ algorithms without troponin testing (Vancouver,^13^ CARE,^23^ and HEAR rules^24^) and even without ECG (Marburg score^25^) have been validated. Our model designed for early prehospital use fits well within the continuum of the current diagnostic approaches.

### Clinical implications: portability and decision-time reduction

A central implication of this work is feasibility of implementing a clinically meaningful ACS risk assessment within a compact, patient-operated device. Despite decades of effort and major improvements in in-hospital processes, total symptom-to-treatment time in ACSs has not meaningfully shortened,^2^^,^^3^ as it largely depends on patient symptom recognition and access to the health care system. In practical terms, such a system could be used by patients at home or in other nonclinical settings at the onset of chest pain, before contacting emergency medical services or presenting to the emergency department. By integrating symptom and baseline risk factors with ECG changes, it could support earlier risk stratification and help guide the urgency of response. In addition, transmitted HB-ECG, PER, and SR data could provide structured decision support for a remotely located physician in settings where immediate in-person medical evaluation is not readily available. Rather than replacing formal clinical assessment, the intended role of such a system would be to support decision-making before hospital presentation, reduce patient hesitation in higher-risk cases, and potentially provide reassurance in selected lower-risk symptomatic individuals, thereby reducing unnecessary emergency department utilization.

### Endpoint-specific model behavior

Our results indicate that model behavior differs by endpoint in a clinically intuitive manner. As seen in Central Illustration, HB-ECG-only–derived separation is modest for ACS overall but more pronounced for STEMI. This contrast highlights the differential role of ECG features across ischemic phenotypes: ECG findings are central for STEMI detection, whereas ACS discrimination depends more on clinical context.

### Comparison with physician interpretation

Although consensus physician interpretation of a single 12-lead ECGs remained numerically superior to an automatic analysis of a single limited-lead recording, it did not take into account human interobserver variability which was only fair, similar to the level of agreement reported in other studies.^26^ Incorporation of baseline-referenced ECG measurements markedly improved HB-ECG-only performance, approaching that of a human single 12-lead ECG interpretation. Full ACS risk prediction model utilizing clinical pretest probability was at least as good (and possibly better when using postevent referencing) as consensus human clinical triage decision without limitations of human interobserver variability. Importantly, this comparison reflects early triage conditions based on a single ECG with limited clinical information rather than the full emergency department diagnostic process incorporating serial ECGs and biomarker testing.

### Study Limitations

The study has several limitations. First, it was retrospective analysis of a modest size sample, which may limit the precision of model estimates and subgroup analyses. It was a single-center study conducted at a tertiary referral center with a substantially higher regional coronary disease burden than the current estimated prevalence in the United States.^27^ Therefore, for the use in different geographic areas the model might need recalibration using modern ASCVD risk estimators such as SMART2/SCORE2 family.^28^, ^29^, ^30^ External validation in independent cohorts will be required to confirm the generalizability of the proposed diagnostic algorithm.

STEMI event counts were low, precluding STEMI model performance assessment beyond establishing of the predominant role of the STM.

The postevent reference ECG represents an imperfect proxy for a true preevent baseline, as it can be affected by interval clinical events and persistent post-ACS changes, and requires further study.

HB-ECG recordings were obtained at the emergency department presentation rather than at symptom onset. Given the dynamic evolution of ischemic ECG changes, diagnostic thresholds may be different earlier in the course of ACS.

The current analysis used a hybrid implementation in which the HeartBeam device was used only to record HB-ECG signals, whereas the PER and SR components were obtained by study physicians rather than by patients through the system interface. Although PER variables could potentially be obtained before device use, for example, through integration with electronic medical records, the ability of patients to reliably self-record ECGs and complete symptom assessments during an acute chest pain episode remains uncertain. Patient training, similar to that required for consumer ECG-enabled devices, as well as user interface testing and optimization, may therefore be necessary to ensure safe and effective real-world use.

Alternative life-threatening causes of chest pain (eg, pulmonary embolism, aortic dissection) were not incorporated into the diagnostic model.

The human clinical triage decision-making performance used in comparison testing might have been underestimated due to the lack of personal contact with the patients (but could represent the performance of a remotely located physician).

### Future directions and clinical implications

This study represents proof of concept rather than a finalized diagnostic algorithm. Each of the clinical component models (PER and SR) can be improved by incorporating additional baseline clinical information and expanding risk assessment to include alternative high-risk causes of chest pain such as pulmonary embolism and aortic dissection.

ECG-based performance can also be enhanced through the use of true multiple baseline recordings and more advanced ECG analysis such as ST-T vector loop morphology analysis, artificial intelligence methods^31^ in particular for detection of occlusive myocardial infarction^32^ rather than classic STEMI. This would improve identification of patients requiring emergent revascularization. Additional physiologic sensors (blood pressure, pulse oximetry, and temperature) may further improve diagnostic performance.

Prospective studies of a fully integrated system in high-risk patient populations will be required to confirm the clinical utility of the presented algorithm.

## Conclusions

Integration of clinical risk factors, symptom characteristics, and vectorcardiographic analysis of three-lead self-recorded ECGs in a hierarchical fusion model suitable for implementation in a portable, patient-operated device may enable clinically meaningful ACS risk stratification at the early stages of chest pain. Such system could help address an important gap in chest pain management by supporting patient decision-making at symptom onset. This approach could shorten symptom-to-treatment time for high-risk patients while providing reassurance to low-risk symptomatic “worried well” individuals and potentially reducing unnecessary emergency department utilization.

Perspectives **COMPETENCY IN CLINICAL CARE AND PROCEDURAL SKILLS:** An integrated risk prediction approach combining pre-existing cardiovascular risk, symptoms, and portable ECG analysis can accurately stratify patients with chest pain for ACS. Integrating it into portable self-assessment tool with a patient-operated, cable-free ECG device potentially can help shorten symptom-to treatment times in ACSs.

**TRANSLATIONAL OUTLOOK:** Prospective clinical trials evaluating portable, patient-operated ECG device that integrate clinical risk factors with automated ECG analysis are needed to determine its impact on patient behavior, symptom-to-decision time, and downstream clinical outcomes in ACS.

## Funding support and author disclosures

This study received limited support from HeartBeam Inc, including provision of HeartBeam devices, donation of ECG machines to participating institution, and modest physician compensation for study-related activities. Dr Shvilkin received consulting fees, travel support (conference attendance and site visit inspection), and stock ownership from HeartBeam Inc. Dr Zdolšek received consulting fees from HeartBeam Inc. Dr Zlatić received consulting fees and study-related compensation from HeartBeam Inc. Dr Jelić received study-related compensation from HeartBeam Inc. Dr Atanasoski received consulting fees and stock options from HeartBeam Inc. Dr Miletić received consulting fees and stock ownership from HeartBeam Inc. Dr Bojović received consulting fees and stock ownership from HeartBeam Inc. Dr Hadzievski received consulting fees and stock ownership from HeartBeam Inc. Dr Zimetbaum received consulting fees from HeartBeam Inc. Dr Gibson is Chair of the Scientific Advisory Board of HeartBeam Inc. Dr Vukčević received consulting fees and stock ownership from HeartBeam Inc.
