# Supplementary material for: Acute Coronary Syndrome Risk Prediction Using Portable Cable-Free ECG Device Combined With Clinical Risk Assessment
Source: JACC Adv. 2026 May 21;5(6):102827. doi: 10.1016/j.jacadv.2026.102827 (PMC13199771; doi:10.1016/j.jacadv.2026.102827)
Supplement: Supplemental Material [file mmc1.pdf]

Supplement Tables and Figures

Contents

Contents **1**

Supplement Tables **2**

Supplement table 1. Study inclusion and exclusion criteria 2

Supplement Table 2. Symptom Risk (SR) Questionnaire and Scoring:* 4

Supplement Table 3. Univariate odds ratios (OR) of symptoms characteristics 7

Supplement table 4. Univariate odds ratios (OR) of pre-existing ASCVD risk factors (PER) 13

Supplement Table 5 Collinearity assessment of SR and PER final model predictors 18

Supplement Table 6. Outcome distribution by availability of post-event reference HB-ECG 20

Supplement Table 7. Interval clinical events in post-event reference ECG cohort 21

Supplement Table 8. STD Distribution by ACS status and follow-up event categories 23

Supplement Table 9. AUC STD and STM comparison by follow-up event status (post-event ... 25

Supplement Table 10. Permutation analysis: AUC of true and random STD 27

Supplement Table 11. Permutation analysis: AUC Differences between true and random 28

Supplement Table 12. Human ECG interpretation and variability (ACS) 29

Supplement Table 13. Human clinical triage assessment (ACS, test set) 31

Supplement Figures **32**

Supplement Figure 1. Heat map of symptom risk (SR) internal collinearity 32

Supplement Figure 2. Heat map of risk factors (PER) internal collinearity 33

Supplement Figure 3. Heat map of SR/PER cross-domain correlations (color scale 34

Supplement Figure 4. Receiver operating characteristic (ROC) and calibration plots for 36

Supplement Figure 5. Receiver operating characteristic (ROC) and calibration plots 37

Supplement Figure 6. Receiver operating characteristic (ROC) and calibration plots for 38

Supplement Figure 7. AUC (panel A) and calibration (panel B) for PER-only STEMI 39

Supplement Figure 8. Calibration curves for ACS fusion models incorporating 40

Supplement Figure 9. Permutation Analysis of Post-Event Reference ECG Assignment 41

Supplement Figure 10. Heat map of pairwise inter-observer agreement among 5 human 42

Supplement Tables

Supplement table 1. Study inclusion and exclusion criteria

Inclusion Criteria:
Males and females older than 21 years who presented to Emergency Department with non-traumatic chest pain that warranted ACS workup and provided informed consent.
Exclusion Criteria:
• Clear alternative diagnosis<br>• Unable or unwilling to provide informed consent<br>• Unable to achieve a diagnostic quality ECG or HeartBeam recording (tremor, agitation)<br>• Uninterpretable ECG due to ventricular pacing or left bundle branch block<br>• Atrial fibrillation or flutter limiting ST-segment measurements<br>• Participation in another trial of an investigational drug or device within 30 days<br>• Other medical or psychological conditions which in the investigators' opinion may increase risk to the patient, raise questions regarding validity of informed consent, or otherwise may interfere with the patient's optimal treatment (for example, acute intoxication, delirium or severe cognitive impairment, or hemodynamic instability requiring immediate intervention).

ECG = electrocardiogram; **HeartBeam** = portable three-lead ECG recording device used in this study.

Supplement Table 2. Symptom Risk (SR) Questionnaire and Scoring:*

List of questions

	Pain assessment dimension	Pain characteristic	Score
**1.1**	Location: Exclusive	Substernal	+3
1.2		Precordial	+2
1.3		Neck, Jaw, Epigastrium	+1
1.4		Left chest	-1
1.5		Right chest	+1
1.6		Back	0
1.7		Other	0
2.1	Pain area size: Exclusive	Less than an inch	-1
		More than an inch	+1
**3.1**	Radiation: Exclusive	Left or right arm	+2
3.2		Both shoulders or right shoulder	+3
3.3		Left Shoulder, or back, or neck, or lower jaw	+1
3.4		Abdomen, or lower back	-1
3.5		Other	0
3.6		None	0
**4.1**	Characteristics: Exclusive	Crushing, pressing, squeezing	+3
4.2		Heaviness, tightness	+2
4.3		Burning/aching	+1

4.4**	Only for pts with CHD, angina	Similar or worse than previous angina	+3
4.5		Sticking, stabbing, catching, pinprick	-1
4.6		Other	0
**5.1**	Aggravated by: Exclusive	Gets worse with exertion, makes you stop/slow down	+2
5.2		Gets worse with deep breath, cough	-1
		Constant, not changing	0
**6.1**	Associated symptoms: Additive	Nausea or vomiting<br>Cough<br>Coughing up blood	+2
6.2		Shortness of breath	+2
6.3		Sweating	+3
6.4		None	0
**7. 1**	Duration: Exclusive	Intermittent (momentary) or < 2 min at a time	-1
7.2		2-15 min	1
7.3		15-60 min	1
7.4		60 min - 12 hrs	0
7.5		> 12 hrs	-1
**8. .1**	Frequency: Exclusive	>=2 per 24 hrs	1

8.2		1 or less per 24 hrs	0
**9. 1**	Additional characteristics:<br>Exclusive	Reproduces or worsens by palpation	-1
9.2		Not sensitive on palpation	0

Exclusive - only single answer

Additive - multiple answers

SR = symptom risk; **CHD** = coronary heart disease; **PE** = pulmonary embolism.

Exclusive = only one response may be selected; **Additive** = multiple responses may be selected.

* Symptom scoring was initially planned as an additive risk model but was replaced by logistic regression because of suboptimal performance.

** 4.4 Might be chosen with one of the other options of group 4.

Additional non-scored items added for future use to assess probabilities of pulmonary embolism and aortic dissection.

Supplement Table 3. Univariate odds ratios (OR) of symptoms characteristics

		Learning set (n = 96)							Test set (n = 88)						
Chest pain dimension		OR	CI (95%)	ACS+		ACS–		p	OR	CI (95%)	ACS+		ACS–		p
				+	-	+	-				+	-	+	-	
Location	Substernal	2.88	0.89–9.30	23	4	46	23	0.08	1.17	0.37–3.74	11	5	47	25	0.79
	Left Chest	0.83	0.24–2.83	4	23	12	57	0.76	0.75	0.19–2.93	3	13	17	55	0.68
	Right Chest	0.00		0	27	6	63	1.00	0.00		0	16	2	70	1.00
	Epigastrium	0.00		0	27	2	67	1.00	2.33	0.20–27.4	1	15	2	70	0.50
	Neck	0.00		0	27	1	68	1.00	0.00		0	16	0	72	1.00
	Precordial	0.00		0	27	2	67	1.00	2.33	0.20–27.4	1	15	2	70	0.50

	Other	0.00		0	27	0	69	1.00	0.00		0	16	0	72	1.00
	Back	0.00		0	27	0	69	1.00	0.00		0	16	2	70	1.00
Radiation	None	0.61	0.22–1.73	6	21	22	47	0.35	0.56	0.16–1.90	4	12	27	45	0.35
	Left Shoulder, Back, Neck, or Lower Jaw	0.80	0.29–2.18	7	20	21	48	0.66	1.47	0.45–4.83	5	11	17	55	0.52
	Left Or Right Arm	3.80	1.42–10.1	12	15	12	57	0.01	2.82	0.81–9.84	5	11	10	62	0.10
	Other	0.00		0	27	4	65	1.00	0.62	0.07–5.42	1	15	7	65	0.66
	Abdomen	0.00		0	27	1	68	1.00	0.00		0	16	4	68	1.00
	Lower Back	1.29	0.11–14.8	1	26	2	67	0.84	0.00		0	16	2	70	1.00

	Both Shoulders Or Right Shoulder	0.34	0.04–2.9	1	26	7	62	0.33	0.89	0.10–8.22	1	15	5	67	0.92
Character	Crushing, Pressing, Squeezing	1.74	0.71–4.30	13	14	24	45	0.23	1.32	0.45–3.92	8	8	31	41	0.61
	Heaviness, Tightness	1.16	0.42–3.24	7	20	16	53	0.78	1.27	0.39–4.13	5	11	19	53	0.69
	Heavy Pressure	2.07	0.77–5.54	20	7	40	29	0.15	1.91	0.49–7.37	13	3	50	22	0.35
	Sticking Stabbing Pinprick	0.18	0.04–0.84	2	25	21	48	0.03	0.20	0.02–1.62	1	15	18	54	0.13
	Other	0.00		0	27	3	66	1.00	0.00		0	16	4	68	1.00

	Burning, aching	5.00	1.10–22.6	5	22	3	66	0.04	Inf		1	15	0	72	1.00
	Similar or Worse Than Previous Angina	0.00		0	27	2	67	1.00	Inf		1	15	0	72	1.00
Associated symptoms	None	0.62	0.23–1.66	7	20	25	44	0.34	2.62	0.86–8.01	10	6	28	44	0.09
	Nausea, Vomiting	0.23	0.03–1.87	1	26	10	59	0.17	0.30	0.04–2.50	1	15	13	59	0.27
	Diaphoresis	2.33	0.77–7.08	7	20	9	60	0.13	0.00		0	16	8	64	1.00
	Dyspnea	0.29	0.06–1.36	2	25	15	54	0.12	0.41	0.05–3.48	1	15	10	62	0.42
Aggravated by	Pleuritic	0.62	0.23–1.66	7	20	25	44	0.34	0.32	0.09–1.07	4	12	37	35	0.06

	Constant	2.18	0.86–5.53	18	9	33	36	0.10	1.80	0.60–5.37	9	7	30	42	0.29
	Worse with exertion	0.42	0.09–2.04	2	25	11	58	0.28	3.09	0.66–14.5	3	13	5	67	0.15
Pain Duration	2-15min	0.42	0.09–2.04	2	25	11	58	0.28	0.59	0.12–2.91	2	14	14	58	0.52
	15-60min	1.69	0.55–5.21	6	21	10	59	0.36	1.62	0.38–6.80	3	13	9	63	0.51
	60min-12h	1.62	0.66–3.98	15	12	30	39	0.29	1.86	0.61–5.67	10	6	34	38	0.27
	>12h	0.49	0.15–1.62	4	23	18	51	0.24	0.25	0.03–2.07	1	15	15	57	0.20
Pain Area Size	< 3cm	0.00		0	27	10	59	1.00	0.00		0	16	6	66	1.00
	> 3cm	Inf		27	0	59	10	1.00	Inf		16	0	66	6	1.00

Pain Frequency	1 Or Less Per 24 Hrs	3.52	1.38–9.00	18	9	25	44	0.01	1.06	0.35–3.26	6	10	26	46	0.92
	> 2 Per 24 Hrs	0.28	0.11–0.73	9	18	44	25	0.01	0.94	0.31–2.89	10	6	46	26	0.92
Reproducible	Not sensitive on palpation	3.77	0.80–17.6	25	2	53	16	0.09	2.51	0.52–12.0	14	2	53	19	0.25
	Reproduces or worsens by palpation	0.27	0.06–1.24	2	25	16	53	0.09	0.40	0.08–1.92	2	14	19	53	0.25
Pain intensity	0 to 10 (per point)	1.45	1.13–1.86					<0.01	1.09	0.84–1.42					0.52

ACS = acute coronary syndrome; **CI** = confidence interval; **OR** = odds ratio.

Supplement table 4. Univariate odds ratios (OR) of pre-existing ASCVD risk factors (PER)

	Learning set (n = 96)							Test set (n = 88)						
Pre-existing ASCVD risk factors	OR	CI (95%)	ACS+		ACS–		p	OR	CI (95%)	ACS+		ACS–		p
			+	-	+	-				+	-	+	-	
Age <40	0.12	0.01–2.06	0	9	27	60	0.142	0.13	0.01–2.37	0	13	16	59	0.170
Age 40–50	0.89	0.29–2.78	5	14	22	55	0.845	1.05	0.26–4.21	3	13	13	59	0.948
Age 51–60	1.85	0.63–5.41	7	11	20	58	0.264	1.73	0.52–5.74	5	15	11	57	0.372
Age 61–70	1.83	0.73–4.57	12	21	15	48	0.197	2.52	0.81–7.77	7	17	9	55	0.109
Age ≥71	0.49	0.13–1.87	3	14	24	55	0.297	0.28	0.03–2.27	1	14	15	58	0.231

Current smoker	0.94	0.37–2.40	9	24	18	45	0.893	1.97	0.65–6.00	10	33	6	39	0.233
Former smoker	2.22	0.78–6.33	8	11	19	58	0.136	0.75	0.19–2.93	3	17	13	55	0.675
Never smoker	0.61	0.24–1.51	10	34	17	35	0.281	0.52	0.14–2.03	3	22	13	50	0.349
Sex	2.08	0.84–5.20	17	31	10	38	0.115	3.26	1.03–10.3	11	29	5	43	**0.045**
High Cholesterol	0.98	0.40–2.40	12	31	15	38	0.966	1.38	0.46–4.13	7	26	9	46	0.569
BP treatment	0.93	0.38–2.27	14	37	13	32	0.876	1.76	0.55–5.59	11	40	5	32	0.337
DM	1.66	0.57–4.81	7	12	20	57	0.348	3.10	0.78–12.2	4	7	12	65	0.107
CKD	0.35	0.02–6.91	0	3	27	66	0.487	1.44	0.06–37.0	0	1	16	71	0.824

CHD	1.49	0.61–3.63	14	29	13	40	0.385	0.97	0.30–3.11	5	23	11	49	0.957
ASA	2.50	1.01–6.21	15	23	12	46	**0.048**	1.36	0.44–4.22	6	22	10	50	0.590
Angina	0.90	0.34–2.37	8	22	19	47	0.830	0.81	0.23–2.80	4	21	12	51	0.738
MI	1.38	0.49–3.90	7	14	20	55	0.549	1.67	0.46–6.06	4	12	12	60	0.438
Stents	1.20	0.37–3.84	5	11	22	58	0.761	2.67	0.69–10.2	4	8	12	64	0.154
CABG	2.68	0.36–20.	2	2	25	67	0.337			0	0	16	72	
NTG	1.68	0.67–4.26	11	20	16	49	0.270	0.37	0.08–1.78	2	20	14	52	0.216
CHF	0.71	0.14–3.65	2	7	25	62	0.680	0.89	0.10–8.22	1	5	15	67	0.921

Diuretics	1.38	0.49–3.90	7	14	20	55	0.549	0.71	0.14–3.56	2	12	14	60	0.681
Stroke	3.83	0.80–18.4	4	3	23	66	0.094	0.73	0.08–6.55	1	6	15	66	0.781
PVD	0.74	0.19–2.92	3	10	24	59	0.664	1.43	0.35–5.93	3	10	13	62	0.621
DVT or PE	0.62	0.07–5.86	1	4	26	65	0.681	0.85	0.04–18.6	0	2	16	70	0.920
Aortic aneurism	0.83	0.03–21.0	0	1	27	68	0.910	1.44	0.06–37.0	0	1	16	71	0.824
Syncope	0.13	0.01–2.36	0	8	27	61	0.169	0.25	0.03–2.07	1	15	15	57	0.201
ASCVD	1.65	0.66–4.11	17	35	10	34	0.281	0.89	0.29–2.72	6	29	10	43	0.837

ASA = aspirin; **ASCVD** = atherosclerotic cardiovascular disease; **CABG** = coronary artery bypass grafting; **CHD** = coronary heart disease; **CHF** = congestive heart failure; **CI** = confidence interval; **CKD** = chronic kidney disease; **DM** = diabetes mellitus; **DVT** = deep vein thrombosis; **MI** = myocardial infarction; **NTG** = nitroglycerin; **OR** = odds ratio; **PCI** = percutaneous coronary intervention; **PE** = pulmonary embolism; **PVD** = peripheral vascular disease; **TIA** = transient ischemic attack.

Supplement Table 5 Collinearity assessment of SR and PER final model predictors (n = 7)

Predictor	VIF	Tolerance
Pain character: Stabbing/pinprick	1.1764	0.85003
Pain duration score	1.1180	0.89449
Aggravated by cough, deep breathing	1.0882	0.91895
Pain area > 3 cm	1.0841	0.92239
Radiation to right or left arm	1.0463	0.95573
Pain character: Burning	1.0398	0.96172
Pain intensity order (0-10), per scale point	1.0238	0.97672

PER Final Model Predictors (n =5)

Predictor	VIF	Tolerance
Age category	1.2597	0.79384

Aspirin use	1.1984	0.83442
Diabetes	1.0972	0.91139
Smoker	1.0863	0.92058
Sex	1.0216	0.97883

ASA = aspirin; **DM** = diabetes mellitus; **PER** = pre-existing ASCVD risk; **SR** = symptom risk; **VIF** = variance inflation factor.

Supplement Table 6. Outcome distribution by availability of post-event reference HB-ECG tracing

Outcome	Post-event reference ECG (n=98)	No post-event reference ECG (n=86)	p value
ACS	27 (27.6%)	16 (18.6%)	0.166
STEMI	8 (8.2%)	6 (7.0%)	0.789

ACS = acute coronary syndrome; **HB-ECG** = HeartBeam electrocardiogram; **STEMI** = ST-elevation myocardial infarction.

Percentages are column percentages. p values were calculated using Fisher exact test.

The frequency of ACS and STEMI did not differ significantly between patients with and without a post-event reference HB-ECG (ACS 27.6% vs 18.6%, p=0.17; STEMI 8.2% vs 7.0%, p=0.79), indicating no evidence of outcome bias related to availability of post-event reference HB-ECG tracing.

Supplement Table 7. Interval clinical events in post-event reference ECG cohort

Total patients in post-event reference cohort: 98

Event	Count	Percent (%)
CABG	2	2.0
PCI	4	4.1
UA	1	1.0
Angina	5	5.1
Myocarditis	1	1.0
Paroxysmal AF	2	2.0
SVT	1	1.0
CHF admission	4	4.1
HTN urgency admission	5	5.1
Chest pain	18	18.4
Palpitations	2	2.0
Non-cardiac surgery	2	2.0
**No events**	**51**	**52.0**

AF = atrial fibrillation; **CABG** = coronary artery bypass grafting; **CHF** = congestive heart failure; **HTN** = hypertension; **PCI** = percutaneous coronary intervention; **SVT** = supraventricular tachycardia; **UA** = unstable angina.

Supplement Table 8. STD Distribution by ACS status and follow-up event categories

Group	N_total	ACS(+)			ACS(-)			P value* ACS(+) vs ACS(-)
		N	Mean	SD	N	Mean	SD	
Overall	98	27	0.183	0.135	71	0.070	0.042	1.63×10^{-6}
No cardiac events	73	17	0.196	0.158	56	0.067	0.038	1.85×10^{-4}
Any cardiac events	25	10	0.161	0.087	15	0.083	0.054	0.012
No major events	90	22	0.186	0.146	68	0.067	0.037	1.09×10^{-5}
Major events	8	5	0.169	0.077	3	0.153	0.064	0.786
Non-major cardiac events	17	5	0.153	0.104	12	0.066	0.037	0.009

ACS = acute coronary syndrome; **CABG** = coronary artery bypass grafting; **HB-ECG** = HeartBeam electrocardiogram; **PCI** = percutaneous coronary intervention; **STD** = ST-vector difference between event and reference ECG; **UA** = unstable angina.

* p values were calculated using the Wilcoxon rank-sum test.

Event definitions:

Major cardiac events: coronary revascularization (CABG or PCI), unstable angina, myocarditis.

All cardiac events: major cardiac events plus angina, paroxysmal atrial fibrillation, supraventricular tachycardia, hospitalizations related to congestive heart failure, hypertension urgency.

Any events: all recorded follow-up clinical events.

No events: absence of reported follow-up clinical events between the index presentation and the post-event reference HB-ECG acquisition.

Supplement Table 9. AUC STD and STM comparison by follow-up event status (post-event reference HB-ECG cohort - ACS outcome)

Group	N	ACS+	ACS−	STD AUC	STD 95% CI	STM AUC	STM 95% CI	ΔAUC	p
Overall	98	27	71	0.801	0.690–0.897	0.736	0.623–0.838	0.065	0.390
Major cardiac events	8	5	3	0.597	0.133–1.000	0.470	0.000–1.000	0.128	0.782
No major cardiac events	90	22	68	0.789	0.661–0.895	0.758	0.638–0.864	0.031	0.710
All cardiac events	25	10	15	0.814	0.627–0.960	0.740	0.527–0.913	0.074	0.584
No cardiac events	73	17	56	0.772	0.616–0.900	0.734	0.589–0.860	0.038	0.702
Any events	47	15	32	0.798	0.646–0.925	0.711	0.551–0.856	0.087	0.408
No events	51	12	39	0.798	0.624–0.938	0.787	0.641–0.909	0.011	0.893

ACS = acute coronary syndrome; **AUC** = area under the curve; **CI** = confidence interval; **HB-ECG** = HeartBeam electrocardiogram; **STD** = ST-vector difference between event and reference ECG; **STM** = ST-vector magnitude; **ΔAUC** = difference in AUC.

Due to the small number of major cardiac events, “major cardiac events” group has extremely wide Cis making its AUC uninterpretable.

Supplement Table 10. Permutation analysis: AUC of true and random STD

Metric	AUC	95% CI
TRUE STD	0.815	0.713–0.906
RANDOM STD	0.751	0.633–0.859
STM	0.735	0.625–0.837

AUC = area under the curve; **CI** = confidence interval; **STD** = ST-vector difference between event and reference ECG; **STM** = ST-vector magnitude during the index ECG; **TRUE STD** = STD calculated using the patient-matched post-event reference ECG; **RANDOM STD** = STD calculated after random reassignment of post-event reference ECGs across patients.

Pairwise AUC comparisons showed a numerical advantage of TRUE STD compared with RANDOM STD and STM, although these differences did not reach statistical significance.

Supplement Table 11. Permutation analysis: AUC Differences between true and random STD

Comparison	ΔAUC	p value
TRUE STD vs RANDOM STD	0.065	0.257
TRUE STD vs STM	0.080	0.116
RANDOM STD vs STM	0.015	0.757

AUC = area under the curve; **STD** = ST-vector difference between event and reference ECG; **STM** = ST-vector magnitude during the index ECG; **ΔAUC** = difference in AUC.
TRUE STD = STD calculated using the patient-matched post-event reference ECG; **RANDOM STD** = STD calculated after random reassignment of post-event reference ECGs across patients.

ROC analysis demonstrated that TRUE STD provided the highest discriminatory performance for ACS (AUC 0.815), compared with RANDOM STD (AUC 0.751) and STM alone (AUC 0.735). Although pairwise AUC differences did not reach statistical significance, the consistent numerical advantage supports the use of patient-specific reference ECG comparison.

Supplement Table 12. Human ECG interpretation and variability (ACS)

12-1. Human Reader Sensitivity and Specificity

Cohorts: full cohort (N=184; ACS=43, non-ACS=141) and post-event reference cohort (N=98; ACS=27, non-ACS=71).

Reader	full cohort sensitivity	full cohort specificity	post-event reference sensitivity	post-event reference specificity
MD 1	0.674	0.830	0.667	0.845
MD 2	0.977	0.511	0.963	0.577
MD 3	0.814	0.901	0.852	0.873
MD 4	0.953	0.404	1.000	0.408
MD 5	0.953	0.539	0.926	0.620
**Consensus**	**0.953**	**0.617**	**0.963**	**0.634**

12-2. Diagnostic performance (AUC) of individual ECG readers and consensus for ACS (full cohort)

Reader	AUC	95% CI	N
MD1	0.751	0.669–0.828	184
MD2	0.743	0.693–0.789	184
MD3	0.858	0.790–0.915	184
MD4	0.678	0.622–0.730	184
MD5	0.746	0.694–0.796	184
Consensus	0.785	0.728–0.835	184

12-3. Overall ECG inter-observer agreement (Krippendorff's alpha)

Metric	Value
Krippendorff's alpha (5 readers, binary ACS decision)	0.380

12-4. Pairwise Cohen's κ between ECG readers

	MD1	MD2	MD3	MD4	MD5
MD1	1.000	0.360	0.539	0.245	0.418
MD2	0.360	1.000	0.326	0.413	0.809
MD3	0.539	0.326	1.000	0.237	0.320
MD4	0.245	0.413	0.237	1.000	0.414
MD5	0.418	0.809	0.320	0.414	1.000

12-5. ECG Per-reader agreement with others and with consensus

Reader	Mean κ vs other readers	κ vs 5-reader consensus
MD1	0.391	0.528
MD2	0.477	0.781
MD3	0.355	0.443
MD4	0.327	0.582
MD5	0.490	0.814

ACS = acute coronary syndrome; **AUC** = area under the curve; **CI** = confidence interval; **ECG** = electrocardiogram; **κ** = Cohen's kappa; **MD** = physician reader; **N** = number of patients.

Krippendorff's α = overall inter-observer agreement coefficient.

Supplement Table 13. Human clinical triage assessment (ACS, test set)

full cohort: N=88 (ACS=16, no ACS=72); post-event reference: N=47 (ACS=11, no ACS=36)

Sensitivity and false-positive rate (FPR): specificity = 1 - FPR.

**Reader**	**Cohort**	**Sensitivity**	**FPR**	**Specificity**
MD 1	full cohort	1.000	0.472	0.528
MD 2	full cohort	1.000	0.542	0.458
MD 3	full cohort	0.750	0.458	0.542
Consensus	full cohort	1.000	0.458	0.542
MD 1	post-event reference	1.000	0.556	0.444
MD 2	post-event reference	1.000	0.694	0.306
MD 3	post-event reference	0.818	0.500	0.500
Consensus	post-event reference	1.000	0.556	0.444

ACS = acute coronary syndrome; **ECG** = electrocardiogram; **FPR** = false-positive rate; **MD** = physician reader; **N** = number of patients. Specificity = 1 - FPR.

Supplement Figures

Supplement Figure 1. Heat map of symptom risk (SR) internal collinearity (color scale represents Spearman's rho), learning set.

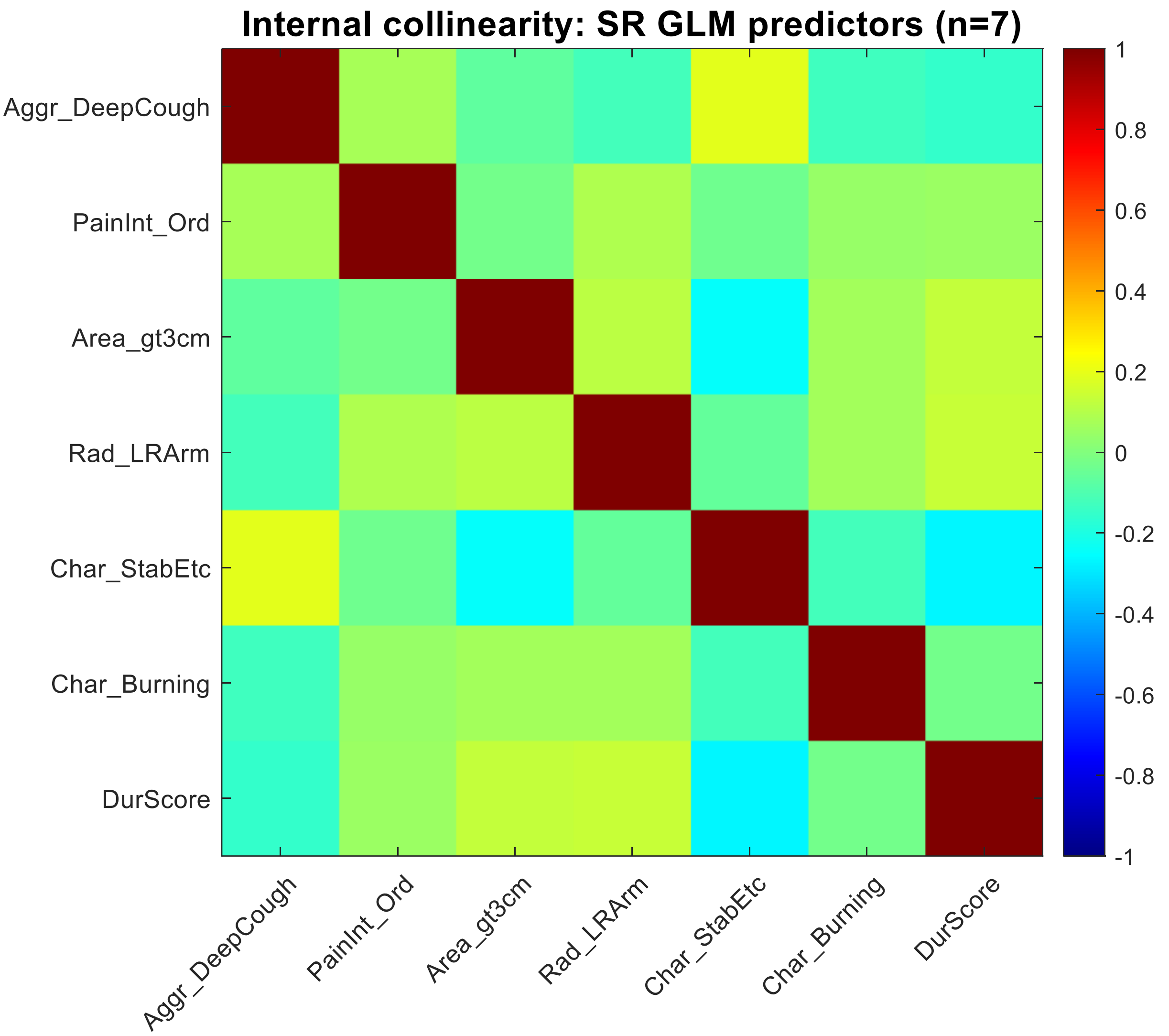

Pairwise correlations among SR predictors were generally low, with no evidence of substantial collinearity.

Abbreviations: Aggr_DeepCough, pain aggravated by deep inspiration or cough; **PainInt_Ord**, pain intensity (ordinal); **Area_gt3cm**, pain area size >3 cm; **Rad_LRArm**, radiation to left or right arm; **Char_StabEtc**, stabbing / pinprick / pleuritic pain quality; **Char_Burning**, burning / aching pain quality; **DurScore**, symptom duration score.

Supplement Figure 2. Heat map of risk factors (PER) internal collinearity (color scale represents Spearman's ρ), learning set (n = 96).

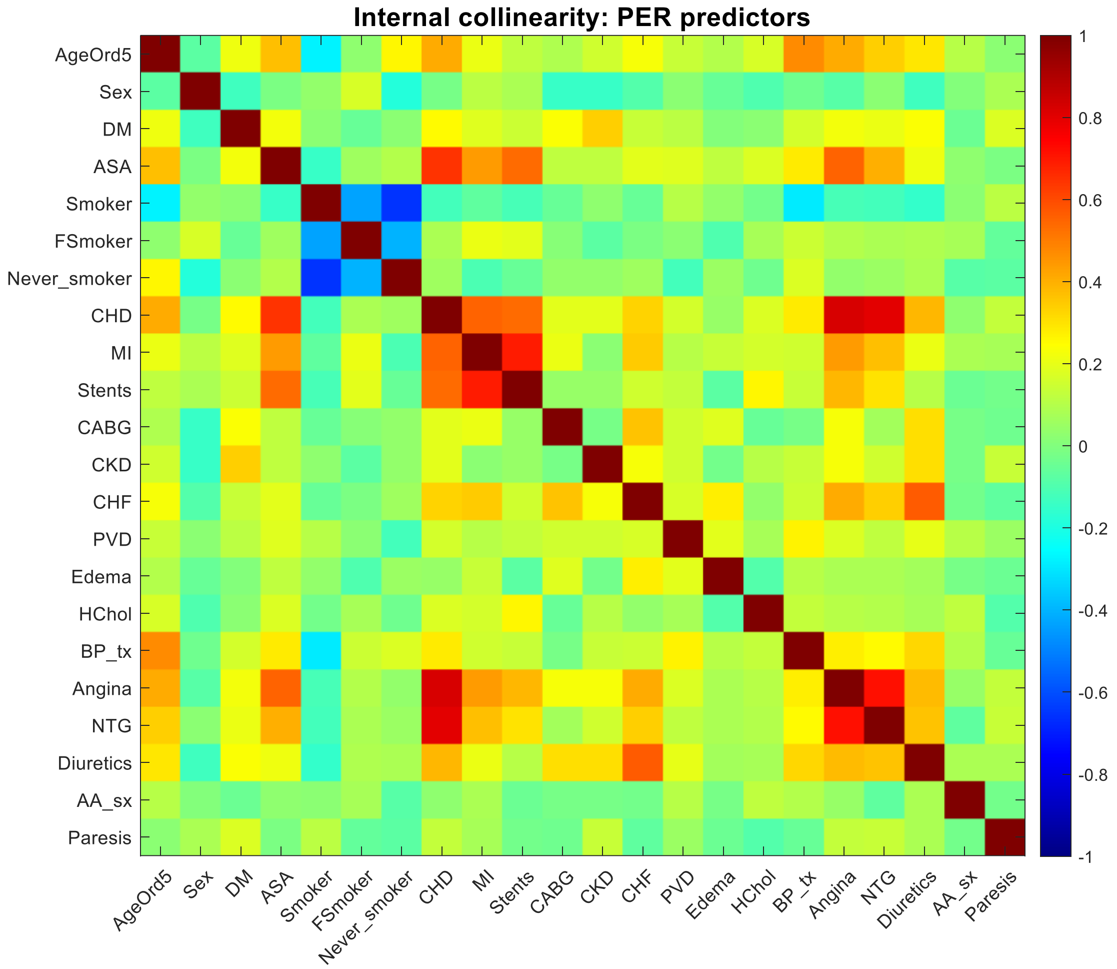

Several expected correlations were observed among clinically related variables, including positive clustering among established coronary disease markers and treatments (e.g. **CHD**, **MI**, **Angina**, **Stents**, **NTG**) and inverse relationships among mutually exclusive categories (e.g. **Smoker** vs **Never_smoker**). These patterns were clinically coherent and reflected overlapping disease burden rather than unexpected collinearity.

Abbreviations: AgeOrd5, age in 5-level ordinal categories; **Sex**, biological sex; **DM**, diabetes mellitus; **ASA**, chronic aspirin use; **Smoker**, current smoking status; **FSmoker**, former smoker; **Never_smoker**, never smoker; **CHD**, coronary heart disease; **MI**, prior myocardial infarction; **Stents**, prior coronary stent implantation; **CABG**, prior coronary artery bypass grafting; **CKD**, chronic kidney disease; **CHF**, congestive heart failure; **PVD**, peripheral vascular disease; **Edema**, peripheral edema; **HChol**, hypercholesterolemia; **BP_tx**, antihypertensive treatment; **Angina**, prior angina; **NTG**, nitrate use; **Diuretics**, diuretic use; **AA_sx**, abdominal aneurism symptoms; **Paresis**, paresis

Supplement Figure 3. Heat map of SR/PER cross-domain correlations (color scale represents Spearman's ρ), learning set (n= 96).

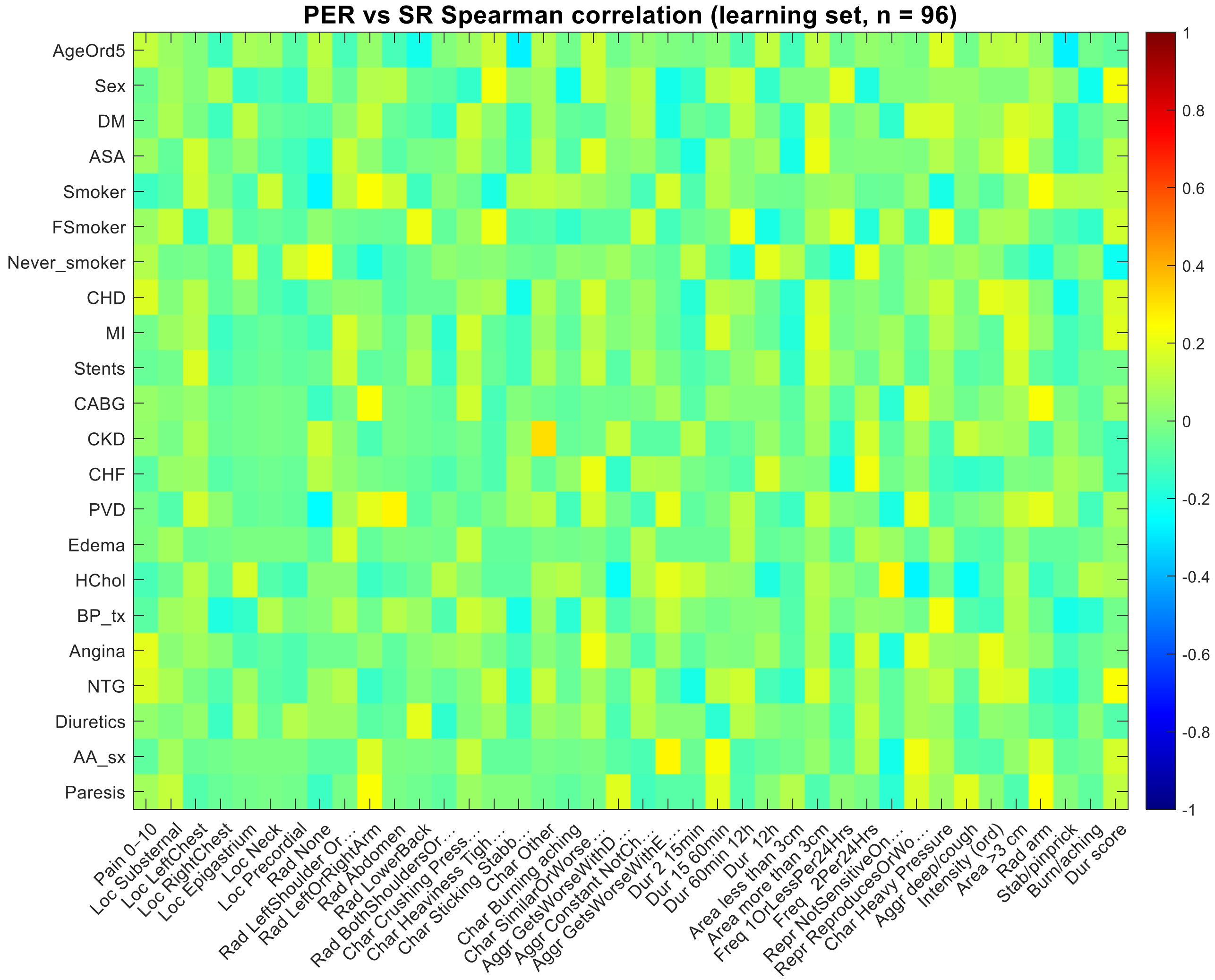

Correlations between pre-existing risk and symptom predictors were generally weak, with no dominant cross-domain clustering, supporting the relative independence and complementary contribution of these predictor domains in the fusion model.

Predictor abbreviations:

Pre-existing risk (PER) predictors

AgeOrd5, age in 5-level ordinal categories; **Sex**, biological sex; **DM**, diabetes mellitus; **ASA**, chronic aspirin use; **Smoker**, current smoking status; **FSmoker**, former smoker; **Never_smoker**, never smoker; **CHD**, coronary heart disease; **MI**, prior myocardial infarction; **Stents**, prior

coronary stent implantation; **CABG**, coronary artery bypass grafting; **CKD**, chronic kidney disease; **CHF**, congestive heart failure; **PVD**, peripheral vascular disease; **Edema**, peripheral edema; **HChol**, hypercholesterolemia; **BP_tx**, antihypertensive treatment; **Angina**, prior angina; **NTG**, nitrate use; **Diuretics**, diuretic use; **AA_sx**, aortic aneurism symptoms; **Paresis**, paresis.

Symptom risk (SR) predictors

Pain_0_10, pain intensity on 0–10 scale; **Loc_Substernal**, substernal chest pain location; **Loc_LeftChest**, left chest pain location; **Loc_RightChest**, right chest pain location; **Loc_Epigastrium**, epigastric pain location; **Loc_Neck**, neck pain location; **Loc_precordial**, precordial pain localization; **Rad_None**, no pain radiation; **Rad_LeftShoulderOr…**, radiation to left shoulder or arm; **Rad_RightArm**, radiation to right arm; **Rad_LowerBack**, radiation to lower back; **Rad_BothShouldersOr…**, radiation to both shoulders or arms; **Char_CrushingPressOr…**, crushing / pressure-like pain quality; **Char_HeartSicknessTig…**, "heart-sickness/tightness"-type pain quality; **Stab…**, stabbing-type pain quality; **Char_Other**, other pain quality; **Char_BurningAching**, burning / aching pain quality; **Aggr_SimultMovesWith…**, pain aggravated by simultaneous movement; **Aggr_GetsWorseWithD…**, pain aggravated by deep inspiration or cough; **Aggr_GetsWorseWith…**, pain aggravated by exertion or activity; **Const_Notch…**, constant/no change with respiration/movement; **Dur_2_15min**, duration <15 minutes; **Dur_15_60min**, duration 15–60 minutes; **Dur_60min_12h**, duration 60 minutes to 12 hours; **Dur_gt12h**, duration >12 hours; **Area_less_than_3cm**, pain area <3 cm; **Area_more_than_3cm**, pain area >3 cm; **Freq_1ORLessEpisodesPer24Hrs**, frequency 1 episode per 24 hours; **Freq_2EpisodesPer24Hrs**, frequency ≥2 episodes per 24 hours; **Repr NotSensitiveOn…,** not sensitive on palpation; **Repr ReproducibleOrWo…**, reproducible on palpation or movement; **Char_HeavyPressure**, heavy / pressure-like quality; **Aggr_deeply_bre…**, aggravated by deep breathing; **Intensity (ord)**, ordinal pain intensity (scale 0-10); **Area > 3 cm**, pain area >3 cm; **Rad arm**, radiation to arm; **Stab/pinprick**, stabbing / pinprick quality; **Burn/aching**, burning / aching quality; **Dur score**, duration score.

Supplement Figure 4. Receiver operating characteristic (ROC) and calibration plots for SR-only model of ACS prediction.

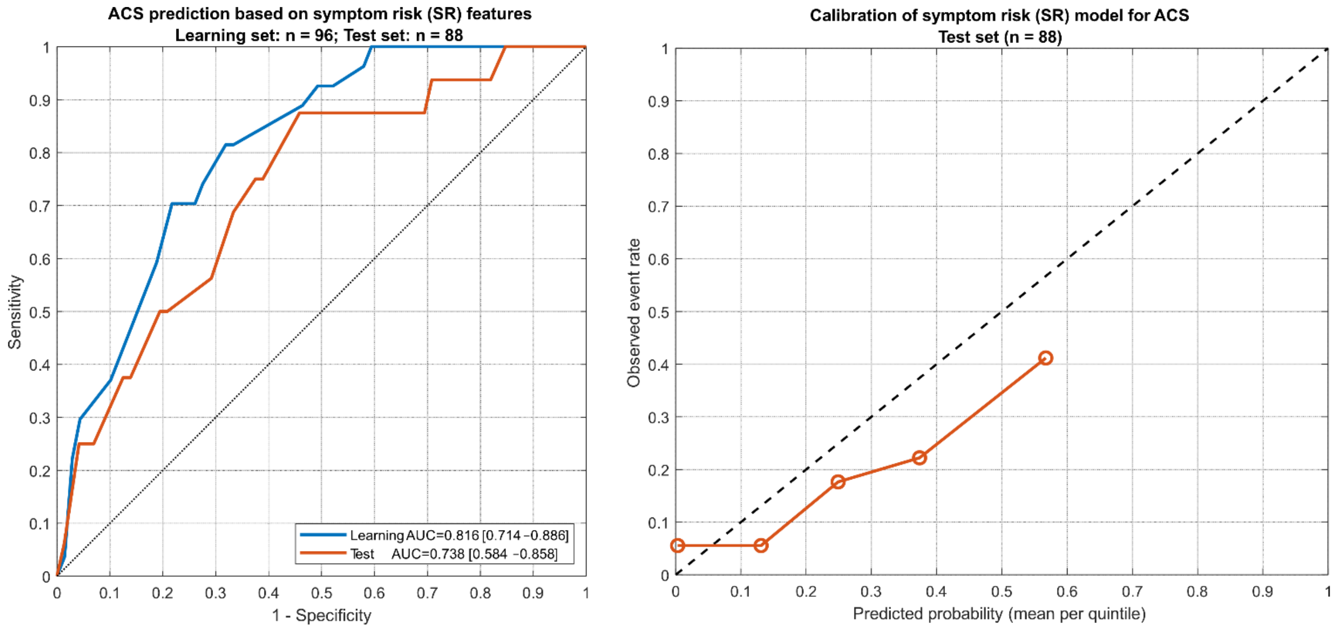

Left panel: learning and test set ROC curves for ACS prediction based on symptom risk features alone.

Right panel: calibration of the SR-only ACS model in the test set, showing observed event rates across quintiles of predicted probability. The ROC plot demonstrates moderate discrimination, and the calibration plot shows reasonable monotonic risk stratification with some underestimation of observed event rates at higher predicted probabilities.

Abbreviations: ACS = acute coronary syndrome; **AUC** = area under the receiver-operating characteristic curve; **ROC** = receiver-operating characteristic; **SR** = symptom risk.

Supplement Figure 5. Receiver operating characteristic (ROC) and calibration plots for SR-only model of STEMI prediction

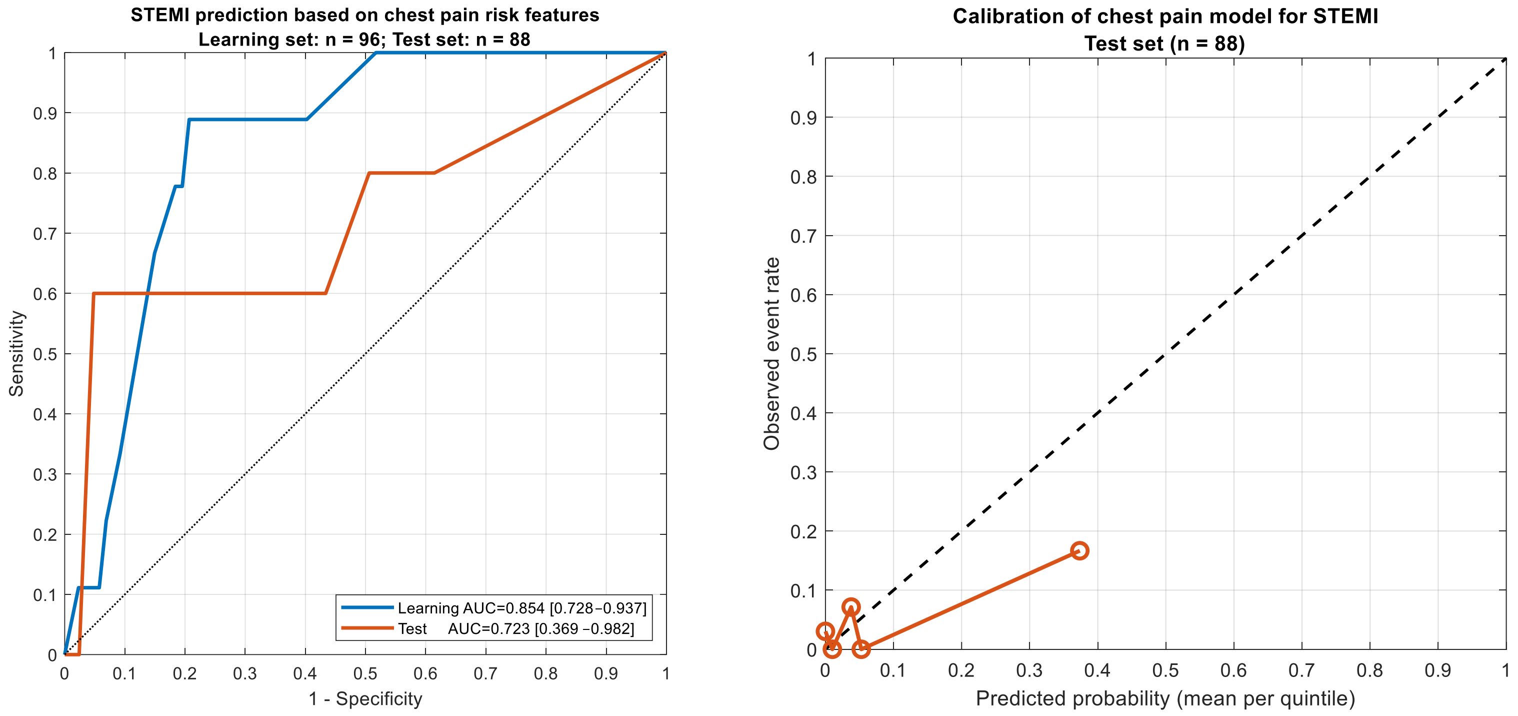

Left panel: learning and test set ROC curves based on symptom risk features alone.

Right panel: test set calibration plot showing observed event rates across quintiles of predicted probability. The model showed fair discrimination with inconsistent calibration, in part due to the small number of STEMI events.

Abbreviations: AUC = area under the receiver-operating characteristic curve; **ROC** = receiver-operating characteristic; **SR** = symptom risk; **STEMI** = ST-segment elevation myocardial infarction.

Supplement Figure 6. Receiver operating characteristic (ROC) and calibration plots for PER-only model for ACS prediction

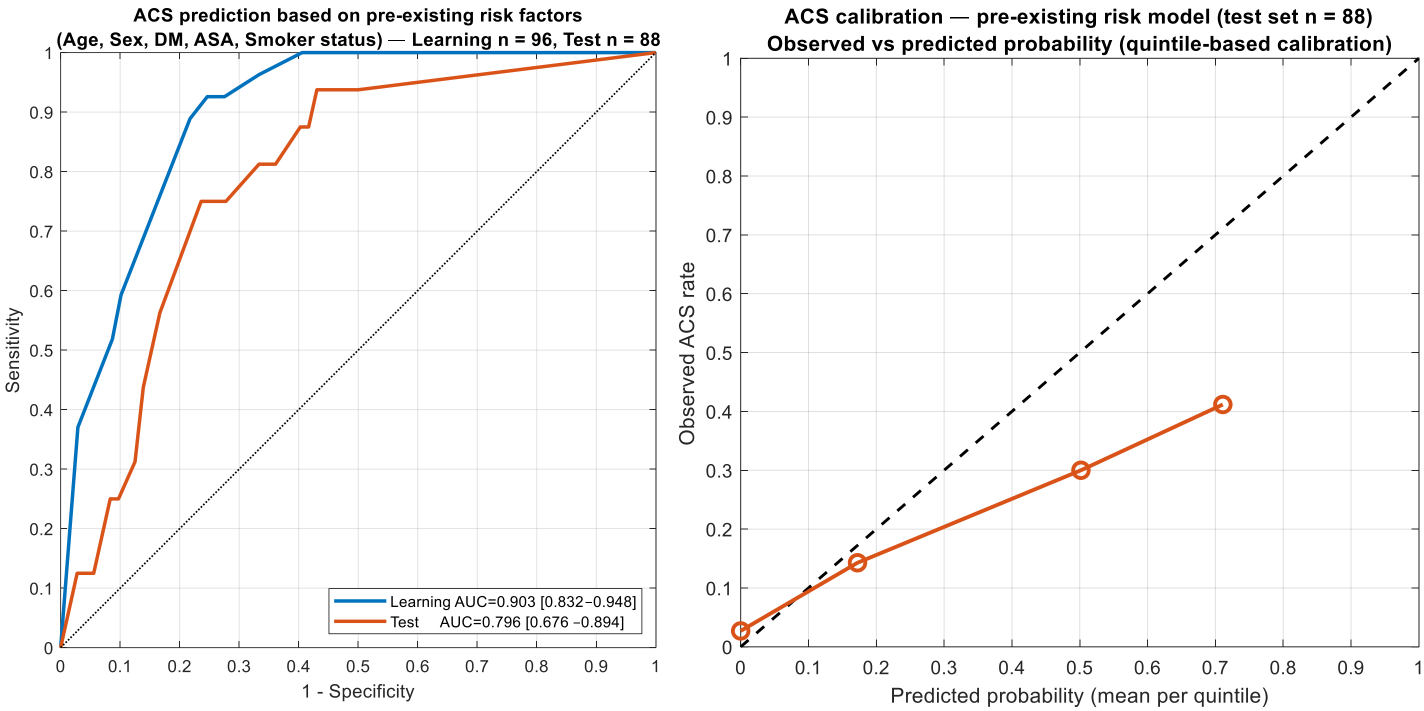

Left panel: learning and test set ROC curves based on pre-existing risk factors alone.

Right panel: test set calibration plot showing observed ACS event rates across quintiles of predicted probability. The model showed good discrimination with moderate attenuation of observed event rates at higher predicted probabilities.

Abbreviations: ACS = acute coronary syndrome; **AUC** = area under the receiver-operating characteristic curve; **PER** = pre-existing risk factors; **ROC** = receiver-operating characteristic.

Supplement Figure 7. AUC (panel A) and calibration (panel B) for PER-only STEMI prediction model

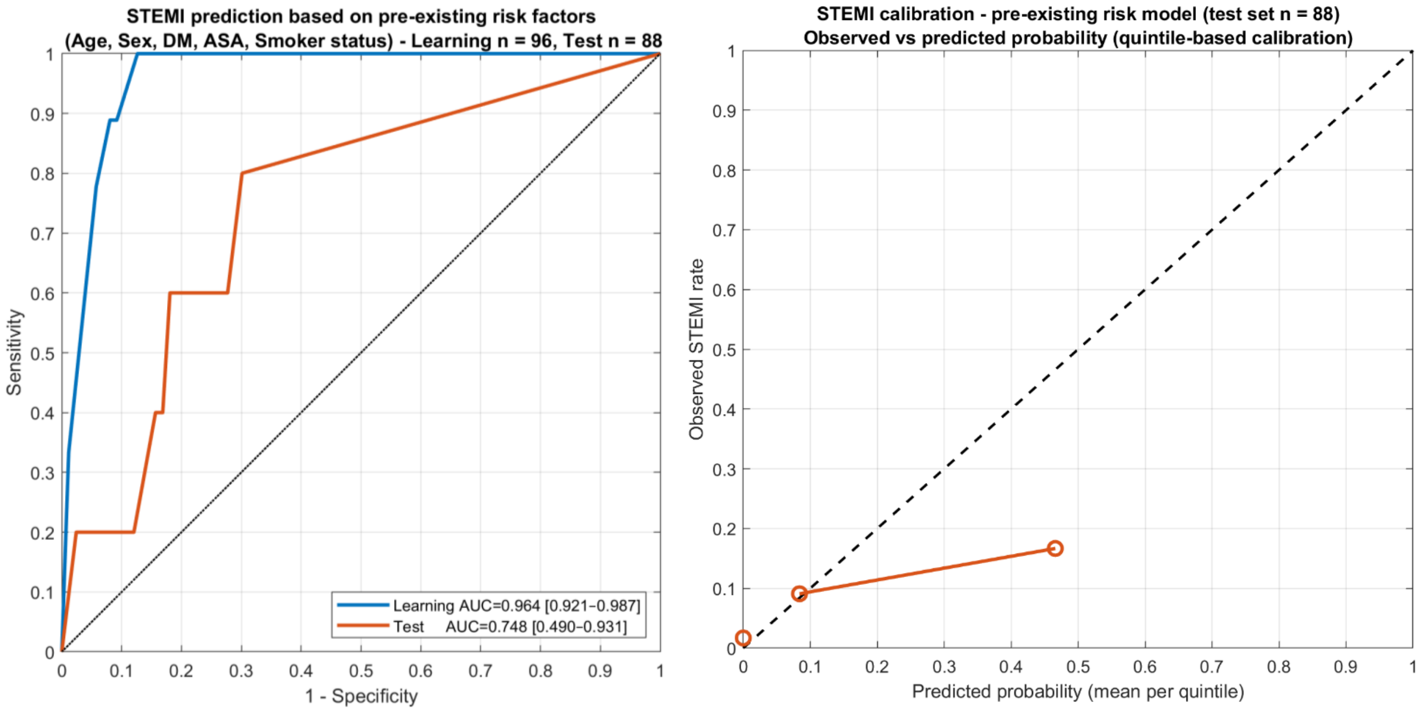

Left panel: learning and test set ROC curves based on pre-existing risk factors alone.

Right panel: test set calibration plot showing observed STEMI event rates across quintiles of predicted probability. The model showed good apparent discrimination in the learning set but more limited and imprecise performance in the test set, with inconsistent calibration in part due to the small number of STEMI events.

Abbreviations: AUC = area under the receiver-operating characteristic curve; **PER** = pre-existing risk factors; **ROC** = receiver-operating characteristic; **STEMI** = ST-segment elevation myocardial infarction.

Supplement Figure 8. Calibration curves for ACS fusion models incorporating PER + SR + STM and PER + SR + STD models

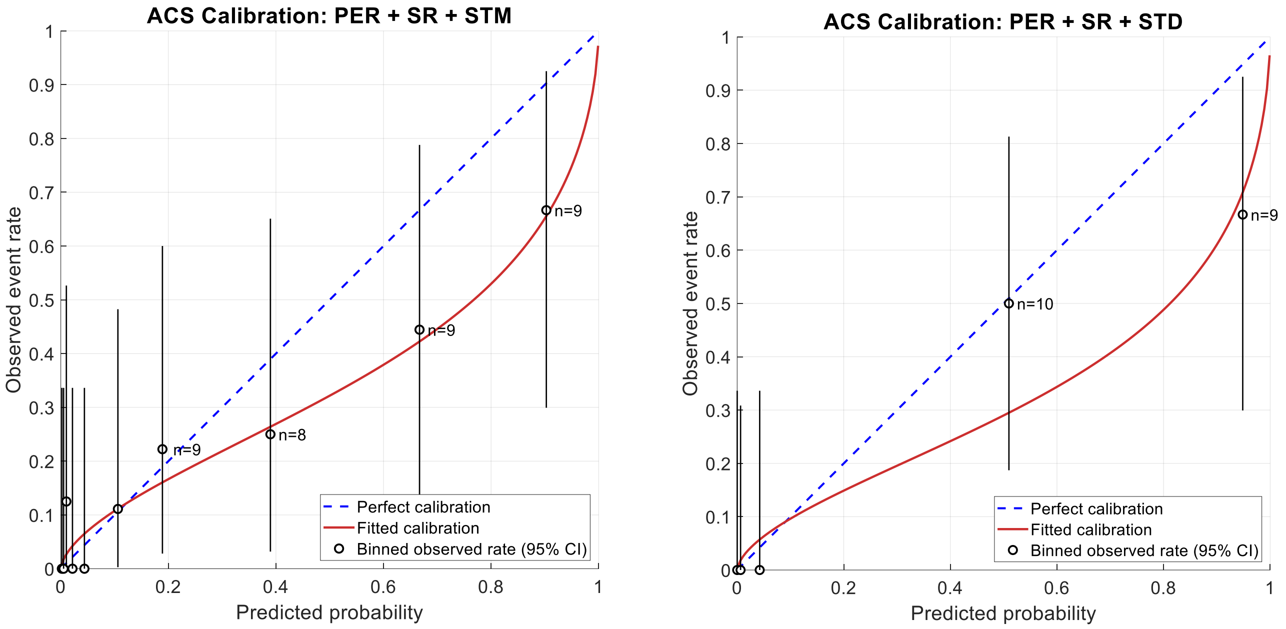

Calibration curves for ACS fusion models:

Left panel: PER + SR + STM model in the full test cohort.

Right panel: PER + SR + STD model in the post-event reference test cohort.

Calibration is shown using observed ACS event rates across binned predicted probabilities, with 95% binomial confidence intervals and fitted calibration curves. In both panels, the fitted calibration curve lies below the line of perfect calibration over much of the higher probability range, suggesting overprediction at higher predicted risks.

Abbreviations: ACS = acute coronary syndrome; **PER** = pre-existing risk factors; **SR** = symptom risk; **STD** = ST-vector difference; **STM** = ST-vector magnitude.

Supplement Figure 9. Permutation Analysis of Post-Event Reference ECG Assignment

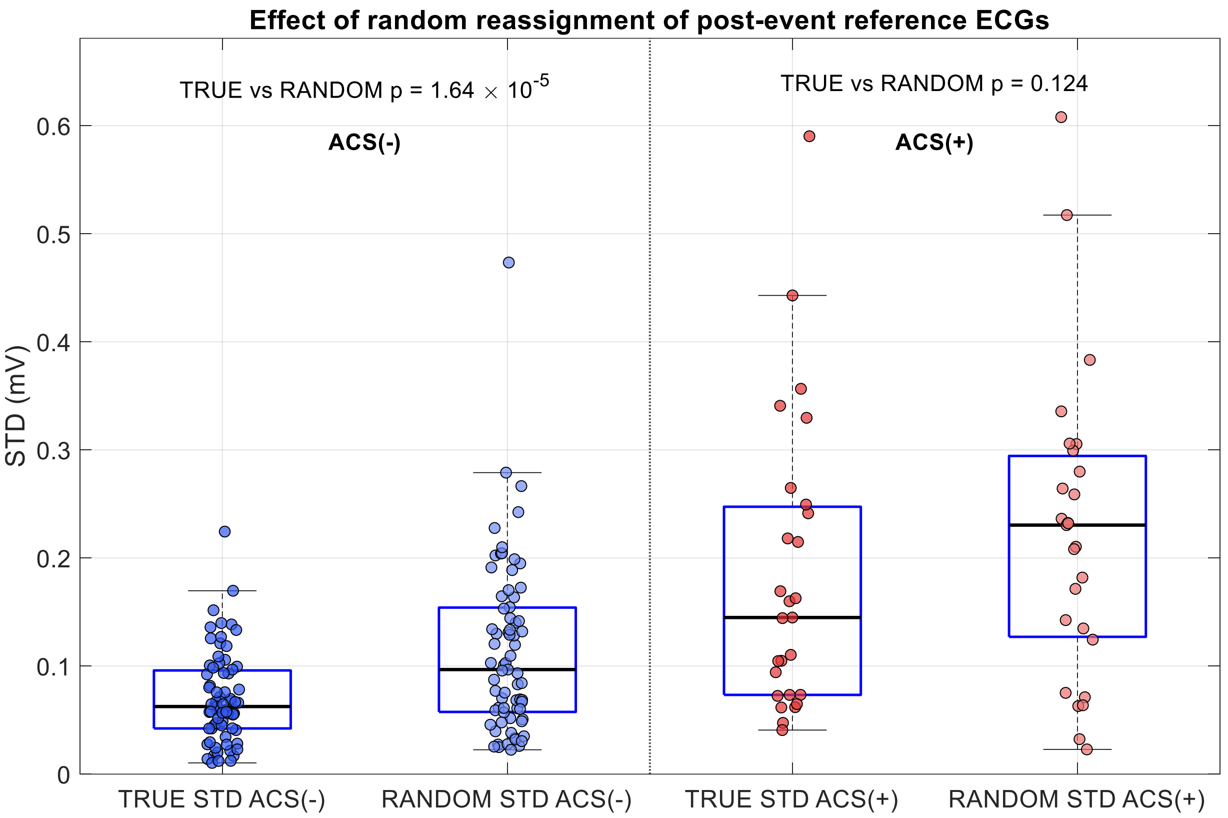

Permutation analysis comparing patient-specific ("true") versus randomly reassigned post-event reference ECGs for calculation of ST-segment shift difference (STD). Left pair, ACS-negative patients; right pair, ACS-positive patients. Box-and-whisker plots with overlaid individual observations are shown for STD values derived using the true post-event reference ECG and after random reassignment of reference ECGs. Boxes indicate the interquartile range with median lines; whiskers extend to 1.5 × the interquartile range. Random reassignment increased STD predominantly in ACS-negative patients, with a significant difference between true and random STD in ACS-negative cases but not in ACS-positive cases, supporting the specificity benefit of patient-specific post-event referencing.

Abbreviations: **ACS** = acute coronary syndrome; **ECG** = electrocardiogram; **STD** = ST-segment shift difference.

.

Supplement Figure 10. Heat map of pairwise inter-observer agreement among 5 human 12-lead ECG readers for ACS classification

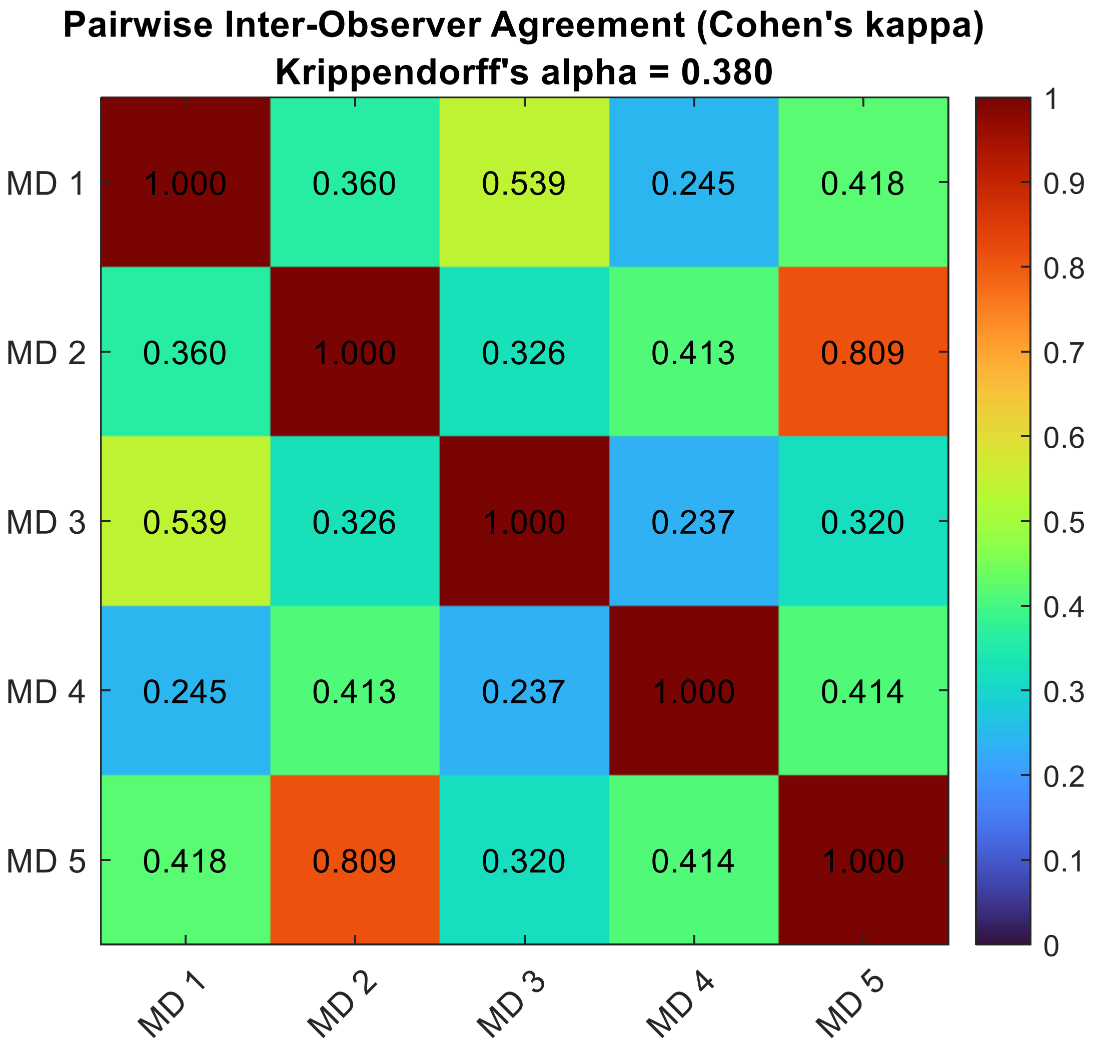

Heat map of pairwise inter-observer agreement among 5 human 12-lead ECG readers (MD 1 – MD 5) for ACS classification, expressed as Cohen's kappa. Overall multi-reader agreement was fair-to-moderate (Krippendorff's alpha = 0.380). Pairwise agreement ranged from fair to substantial, with Cohen's kappa values from 0.237 to 0.809.
